# Generative Artificial Intelligence for Qualitative Methods in Health Research: Rapid Review

**DOI:** 10.2196/98551

**Published:** 2026-08-31

**Authors:** Raúl D Gierbolini-Rivera, Milena Franco Silva, Alexandre Augusto de Paula da Silva, Ross C Brownson, Diana C Parra, Maura M Kepper, Amy A Eyler

**Affiliations:** 1 Prevention Research Center, Bursky School of Public Health, Washington University in St. Louis, One Brookings Drive, St. Louis, MO, 63130, United States, 1 7874294475; 2 Alvin J. Siteman Cancer Center, School of Medicine, Washington University in St. Louis, St. Louis, MO, United States; 3 Brown School, Washington University in St. Louis, St. Louis, MO, United States

**Keywords:** generative AI, qualitative methods, large language models, health, evidence synthesis

## Abstract

**Background:**

Generative AI (GAI) is rapidly transforming research practices, including qualitative methods in health research. While these tools offer efficiency in processing large volumes of textual data, concerns remain regarding their methodological rigor, interpretive capacity, equity, and ethical implications.

**Objective:**

This rapid review aimed to synthesize the current evidence on the use of GAI in health-related qualitative research, focusing on its applications, performance relative to human analysis, and implications for rigor, ethics, and equity.

**Methods:**

We conducted a rapid review following Joanna Briggs Institute and PRISMA (Preferred Reporting Items for Systematic Reviews and Meta-Analyses) guidelines. Peer-reviewed studies published between 2022 and December 2025 were identified through searches in PubMed, Web of Science, and Scopus. Eligible studies included qualitative or mixed methods research that used GAI tools (eg, ChatGPT, Gemini, and Claude) during qualitative analysis, including studies that compared GAI-generated outputs with human researchers, coders, or traditional qualitative analytic approaches. Data were extracted using a structured template and synthesized descriptively. Study quality was assessed using the Critical Appraisal Skills Programme (CASP) checklist. This rapid review was registered with the International Prospective Register of Systematic Reviews (PROSPERO; CRD420261280832). The review adhered to the registered PROSPERO protocol; no deviations occurred.

**Results:**

A total of 42 studies met the inclusion criteria; 71.4% (n=30) were published in 2025, and 81% (n=34) used qualitative designs. Thematic analysis (n=20, 40%) and content analysis (n=10, 20%) were the most common qualitative approaches. GAI was most applied during data familiarization, coding, and theme development, with ChatGPT being the most frequently reported GAI, accounting for nearly two-thirds of all model occurrences (n=37, 62.7%). Among studies evaluating GAI performance relative to human qualitative analysis, performance was strongest in inductive thematic and content analyses, with agreement often exceeding 80% for descriptive themes but dropping to approximately 30% for culturally nuanced themes. Several studies reported time to complete analyses up to 97% faster than human-led analyses. However, performance declined for reflexive and theory-driven analyses, particularly when interpreting culturally nuanced or emotionally complex data. Across studies, GAI improved efficiency but frequently produced superficial interpretations, misapplied theoretical frameworks, and generated occasional inaccuracies, including fabricated quotes. Human oversight was consistently identified as essential to ensure validity, contextual accuracy, and ethical integrity. Concerns related to bias, transparency, and data privacy were widely reported.

**Conclusions:**

GAI can effectively support early-stage qualitative analysis and enhance efficiency in health research; however, it cannot replace the interpretive and reflexive functions central to qualitative inquiry. A hybrid human-AI approach is recommended, in which GAI assists with data processing while researchers retain responsibility for interpretation, contextualization, and ethical oversight. Future research should prioritize developing guidelines that address equity, transparency, and responsible integration of GAI into qualitative methodologies.

## Introduction

Qualitative research is foundational in public health, health policy, and the social sciences because it explores experiences, meanings, processes, and contexts that shape health using nonnumerical data [1,2]. Common data collection methods include interviews, focus groups, participant observation, and textual analysis [1,2]. With flexible and iterative designs, these methods enable researchers to understand how people make health-related decisions and interpret the symbolic meanings embedded in their words and actions [1]. This reflexive process can also be applied to existing documents, such as policies, media texts, social media posts, and legislation, to uncover contextual influences and intentions. Major methodological approaches include thematic analysis (identifying themes), grounded theory (generating theory from patterns), framework analysis (applying structured models), content analysis (categorizing text), and ethnography (describing cultures and social worlds) [1,2]. Qualitative research identifies barriers and facilitators to health, informs intervention development, strengthens mixed methods designs, and deepens understanding of the social and contextual forces shaping health behaviors and outcomes [1].

The rapid emergence of generative AI (GAI) has accelerated its use in qualitative research [3]. GAI refers to a subset of AI that creates new content by learning patterns from existing data [1]. Tools such as ChatGPT (OpenAI), Claude (Anthropic), and Gemini (Google) are transformer-based large language models (LLMs), a type of GAI trained on large volumes of text data that can generate human-like, semantically coherent responses and process large qualitative datasets [1,4]. Unlike traditional machine learning models focused on prediction or classification, GAI produces novel outputs, including text, code, or images, based on statistical pattern learning [5]. These tools can identify themes, sentiments, and trends; generate coding schemes; transcribe interviews; analyze audio or video data; and even support structured approaches such as grounded theory [1]. However, the literature highlights important limitations. GAI systems also struggle with subcultural slang and culturally specific discourse, posing concerns for global and equity-focused public health research [1]. Jowsey et al [6] argue that GAI lacks true reflexive capacity because it relies on statistical prediction rather than genuine comprehension of meaning, context, and human experience. Transformer-based models frequently hallucinate, generate nonexistent information, reproduce training data biases, and pose privacy risks when used in cloud-based systems [1,4].

**Figure 1.**
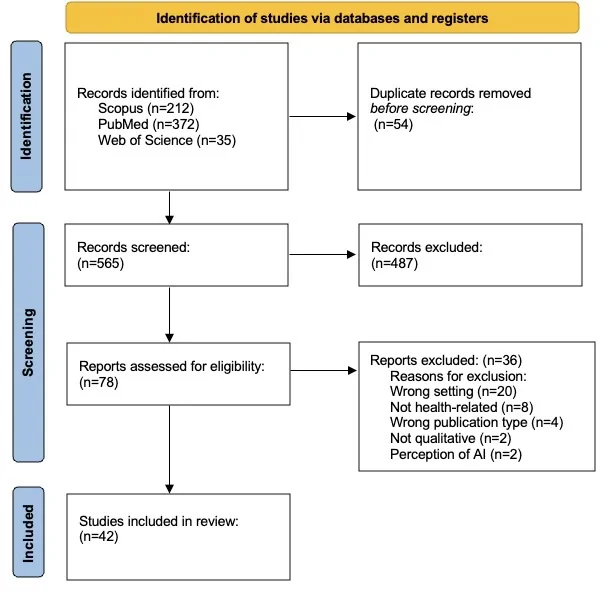
PRISMA (Preferred Reporting Items for Systematic Reviews and Meta-Analyses) flowchart of the included studies (n=42).

GAI tools are integrated into multiple stages of qualitative research. AI-enabled computer-assisted qualitative data analysis software (CAQDAS) platforms (NVivo, ATLAS.ti, and MAXQDA) and general-purpose LLMs (ChatGPT, Claude, Gemini, and Bard) are used to automate transcription, generate word and concept clouds, auto-code transcripts, produce summary themes, cross-reference codes, and create tables and figures [1]. Researchers have also applied prompt engineering strategies, such as explicit task instructions, contextual background, output templates, role-based prompting, and transparency directives, to improve LLM performance in thematic analysis [4]. Zhang et al [4] found that guided, transparent prompting increased researchers’ trust and shifted attitudes from skepticism to cautious acceptance through iterative refinement. Performance outcomes across different GAI tools, however, are mixed. For example, one study by Prescott et al [7] found that themes generated by GAI (ChatGPT and Bard, now called “Gemini”) were consistent with 71% of themes produced by human analysts following inductive thematic analysis. However, consistency dropped to 50% to 58% for deductive thematic analysis, with intercoder reliability ranging from fair to moderate. Sakaguchi et al [8] reported over 80% agreement on descriptive themes using ChatGPT-4, but only approximately 30% for culturally nuanced themes. Overall, GAI tools help accelerate coding and early theme development but still lack the contextual sensitivity and interpretive depth required for high-quality qualitative analysis [1].

Concerns about methodological rigor and transparency are growing. Monforte [9] warns that GAI tools may reduce qualitative inquiry to pattern recognition, prioritizing speed over reflection. LLMs are seen as “black boxes,” conflicting with qualitative research’s transparency, reflexivity, and reliability [9]. Studies warn that reliance on GAI could undermine deep thinking, critical reading, and reflexivity, raising questions about whether AI can genuinely interpret data or reinforce biases [10-12]. Sigman and Bilinkis [13] call this “cognitive sedentarism,” where critical thinking declines and GAI outputs are accepted uncritically. Ethical and privacy issues are significant; cloud-based AI tools may store data externally or use it for training, risking confidentiality and participant rights [14]. Broader social issues, such as exploitative labor and ecological impacts, complicate GAI use [14]. Universities are creating governance frameworks, such as the University of Texas at Austin’s pilot of Grammarly’s AI with data protections and its “Faculty Guide,” and the University of Central Florida’s AI conference, which brings together higher education professionals to share best practices, ethical considerations, and pedagogical strategies for integrating AI into academia [15]. Vanderbilt University’s “walled-garden” AI systems keep inputs within institutional infrastructure. These measures aim to balance innovation with ethics, privacy, and integrity [15].

Since ChatGPT’s public release in late 2022, the literature has emphasized the need for human insight, interpretation, and validation in qualitative research using GAI. Many recent studies have directly evaluated GAI-generated outputs against human qualitative researchers or traditional qualitative analytic approaches, creating a growing evidence base regarding the strengths and limitations of GAI-assisted qualitative analysis. However, some scholars argue that GAI conflicts with reflexive methodologies that require meaning-making and interpretive rigor and should be avoided. With evolving GAI technologies and debates about their use, a synthesis of evidence is needed. No systematic review exists on the use of GAI in health-related qualitative methods. As GAI is already affecting health researchers, a rapid review is necessary to provide insights into its applications. This review aimed to (1) describe GAI integration; (2) compare performance with humans or CAQDAS toolscomputer-assisted qualitative data analysis soft; (3) assess rigor, reproducibility, ethics, and equity; and (4) identify gaps, risks, and best practices. Terms such as LLMs and GAI tools are used interchangeably to refer to related concepts. The findings of this study can broaden understanding of the current use of AI in health research, as well as the benefits and trade-offs of using it in qualitative methods, especially amid the increasing integration of AI across various fields, including academia and research.

## Methods

### Overview

We conducted a rapid review of journal articles on GAI in health-related qualitative methods, following Joanna Briggs Institute (JBI) and PRISMA (Preferred Reporting Items for Systematic Reviews and Meta-Analyses) guidelines (Checklist 1) [16]. This review was registered in the International Prospective Register of Systematic Reviews (PROSPERO; ID: CRD420261280832; National Institute for Health and Care Research, 2026). A rapid review is a form of knowledge synthesis that accelerates the evidence gathering process to inform urgent decision-making, differentiating itself from a systematic review by streamlining specific methodological steps, such as searching fewer databases or limiting gray literature, to produce results in a faster manner [16,17]. Conducting a rapid review was necessary because GAI is being rapidly integrated into qualitative research and may have implications for research rigor.

### Inclusion Criteria

Studies were included if they met the SPIDER (sample, phenomenon of interest, design, evaluation, research type) framework, which is well-suited for qualitative and mixed-methods rapid reviews [18]. The criteria were as follows. First, the eligibility criteria included qualitative or mixed methods studies conducted in health-related settings (public health, health policy, community health, or related social science contexts with clear health relevance). Studies focused purely on clinical contexts (eg, medical transcription using AI) or education sector contexts were excluded. The review included studies from any health domain, such as chronic disease, health behaviors, and noncommunicable diseases, but excluded articles focused solely on clinical applications, such as medical documentation, surgical decisions, and clinical workflows. This exclusion was intentional because a substantial and rapidly growing body of literature already examines the use of GAI to support clinical workflows and health care operations. The aim of this rapid review was to focus specifically on the use of GAI within qualitative health research methodologies and analytic processes, rather than on clinical or operational applications of AI in health care. Second, the phenomenon of interest was empirical use of GAI tools (eg, ChatGPT, Claude, Gemini, and Llama-based models) in at least one qualitative research step. Conceptual papers and reviews were excluded. Third, the study design included qualitative studies, methodological demonstrations, case studies, and mixed methods or other empirical study designs, provided they contained identifiable qualitative components relevant to the review objectives. Fourth, the evaluation included at least one outcome related to the methodological or practical performance of GAI, including comparison with human researchers, coders, traditional qualitative analytic approaches, or outcomes related to transparency, ethics, or equity associated with its use. Fifth, the research type was limited to empirical, peer-reviewed articles. We included studies published since 2022, aligning with the mainstream adoption of GAI, and limited the search to English-language publications. We included studies from 2022 onward to capture the most recent developments in GAI and better understand emerging ethical and equity concerns.

### Search Strategy

We conducted the search across 3 databases (PubMed, Web of Science, and Scopus), finalizing it on December 11, 2025. These databases were chosen because they cover research in health, public health, and the social sciences, aligning well with the aims of this study. A Boolean logic syntax using all keyword combinations was used (see Appendix I in Multimedia Appendix 1). The full search strategy is provided in Appendix II in Multimedia Appendix 2.

### Study Screening

Initially, we screened studies by title and abstract, then conducted full-text screening of those that met the criteria. One reviewer screened all records, while a second reviewer independently sampled 30% of the records. Discrepancies were resolved by consensus among the 2 reviewers. All screening and agreement checks were documented in Rayyan, an online platform for systematic reviews [19].

### Data Extraction and Synthesis

Data extraction was carried out in accordance with the JBI data extraction standards [17]. A structured Excel data extraction template was adapted from the JBI data collection tools. To ensure consistency, the Excel template was pilot-tested by 2 reviewers on a small subset of studies, enabling the team to make refinements. The extracted data included bibliographic details (such as title, author, year, journal, citation, and study aim), health domain, study setting, study design, country, institutional affiliations, qualitative approach, GAI used, specific use of the AI tool, reliability of human versus GAI, evaluation metrics, main findings, and ethics related to the use of GAI in qualitative health research. All data were systematically extracted by one reviewer, with a second reviewer checking a randomized 30% subset. The results were reported in accordance with PRISMA guidelines; the synthesis did not require a minimum number of studies to be included or analyzed. Refer to Appendix III in Multimedia Appendix 3 for the variables used in data extraction. To enhance readability and alignment, we present a series of synthesis tables in the *Results* section that summarize patterns across GAI methods, performance, ethics, and trade-offs in qualitative health research. The full study-level extraction table is provided in Appendix IV in Multimedia Appendix 4.

### Assessment of Quality

All studies were rated for quality using the Critical Appraisal Skills Programme (CASP) checklist [20,21]. For each item, the following categories were used: “Yes,” “Somewhat,” “Can’t Tell,” or “No.” For the last item in the checklist, a narrative judgment of the value of the research was developed for each study. Regardless of the category in which each item was placed, all studies were included in the review, as recommended by the CASP guidelines [20,21].

## Results

### Characteristics of the Included Studies and Main Findings

The systematic search identified 619 records (Scopus: n=212, 34.2%; PubMed: n=372, 60.1%; and Web of Science: n=35, 5.7%), as shown in Figure 1. After removing 54 (8.7%) duplicates, 565 (91.3%) unique records underwent title and abstract screening, during which 487 (86.2%) were excluded. Seventy-eight (13.8%) papers proceeded to full-text review, and 42 (7.4%) met all inclusion criteria for this rapid review. The reasons for exclusion were the wrong setting (n=20, 55.6%), not related to health (n=8, 22.2%), the wrong publication type (n=4, 11%), not qualitative (n=2, 5.6%), and examining only AI perception (n=2, 5.6%).

Table 1 presents that most studies were published in 2025 (n=30, 71.4%). The majority were qualitative studies (n=34, 81%), while the remainder were mixed methods or other study designs that contained identifiable qualitative components. Thematic analysis (20/50, 40%) and content analysis (10/50, 20%) were the most common methods, and in some studies, multiple qualitative approaches were used. The most referenced health domains across the studies were health communication, cancer, COVID-19, medical education, and mental health. Other, less common health domains included substance use, patient safety, climate change, LGBTQ+ health, and multiple clinical specialties.

**Table 1.** Summary characteristics of the included studies.

Variables and categories	Values, n (%)
Year	
2025	30 (71.4)
2024	12 (28.6)
Study design	
Qualitative only	34 (81.0)
Mixed methods	6 (14.2)
Cross-sectional with qualitative elements	1 (2.4)
Experimental and comparative study with qualitative elements	1 (2.4)
Qualitative approach^a^	
Thematic analysis	20 (40.0)
Content analysis	10 (20.0)
Grounded theory	4 (8.0)
Constant comparative method	2 (4.0)
Framework analysis	2 (4.0)
No formal approach—qualitative elements	2 (4.0)
Case study	1 (2.0)
Codebook-based coding	1 (2.0)
Autoethnography	1 (2.0)
Immersion or crystallization	1 (2.0)
Thematic narrative analysis	1 (2.0)
Qualitative comparative analysis	1 (2.0)
Qualitative description	1 (2.0)
Query-based analysis	1 (2.0)
System dynamics or causal loop	1 (2.0)
Iterative thematic inquiry	1 (2.0)
GAI^b^ model used^a^	
Open AI (ChatGPT)	37 (62.7)
Meta Llama	6 (10.2)
Google Gemini	4 (6.8)
Google Gemma	2 (3.4)
Claude	2 (3.4)
Google Flan	2 (3.4)
Microsoft Copilot	2 (3.4)
Perplexity	1 (1.7)
Grok	1 (1.7)
DeepSeek	1 (1.7)
Mistral AI	1 (1.7)
Health domains^a^	
Health communication	4 (9.5)
Cancer	3 (7.1)
COVID-19	2 (4.8)
Medical education	2 (4.8)
Mental health	2 (4.8)
Substance use	2 (4.8)
Other^c^	27 (64.3)

a There was a total of 42 included studies. The variables of “qualitative approach,” “GAI model used,” and “health domains” may exceed 100% because there were multiple approaches in some studies.

b GAI: generative AI.

c In this other category, there were 27 distinct health domains: addiction, asthma, blindness, cardiovascular disease, climate change, clinical practice, emergency medicine, health care, HIV, hospice care, LGBTQ+ health, maternal health, medication management, nursing, nutrition, obesity, ophthalmology, patient safety, pharmacovigilance, public health, sacred moments, school psychology, sleep medicine, social media, surgery, telemental health care, urology, vaccine hesitancy, and well-being.

### Narrative Synthesis

Across the included studies, GAI was used at multiple stages of qualitative analysis, with varying levels of effectiveness depending on the analytic task, methodological approach, and degree of human oversight. Table 2 summarizes how GAI tools were integrated across the qualitative research stages, their typical uses, the human role, and key observations.

**Table 2.** Integration of generative AI (GAI) across qualitative research stages^a^.

Qualitative stage	Typical use of GAI	Human role	Key observations	Reference
Familiarization	Summarization, surface pattern detection	Contextual verification	Across 6 studies evaluating primarily ChatGPT, Copilot, Mistral, and related LLMs^b^, GAI-generated summaries were broadly similar to manual analyses. Four of the 6 studies reported that outputs were primarily descriptive and missed irony, emotional tone, or contextual nuance, while all 6 studies emphasized the need for human verification. Performance appeared strongest for summarization and surface-level familiarization tasks but varied across models when deeper contextual understanding was required.	[22-27]
Inductive coding	First-pass code generation	Code refinement	Across 2 studies evaluating ChatGPT-3.5 and ChatGPT-4, GAI-generated themes were consistent with those identified by human analysts, with agreement exceeding 80% in one study. Both studies concluded that human review remained necessary. While ChatGPT demonstrated strong first-pass coding capability, evidence was insufficient to determine whether comparable performance would be achieved across other GAI models.	[7,28]
Deductive coding	Codebook application	Error correction	One study (1/2) evaluating Llama 3 70B reported high accuracy for binary and concrete codes but weaker performance on behavioral and interpersonal constructs, including frequent false positives. Both studies recommended hybrid human-AI workflows to improve accuracy, contextual interpretation, and bias mitigation. Findings suggest stronger performance for structured deductive coding than complex interpretive coding tasks.	[22,29]
Theme development	Code clustering	Theoretical interpretation	Across 4 studies evaluating various variants of ChatGPT, and Mistral, GAI-generated themes were broadly similar to those identified through manual analysis. Three of 4 studies reported themes more descriptive and less interpretive than those produced by humans, 3 of 4 studies reported forced or misapplied theoretical interpretations. These limitations were observed across multiple models rather than being unique to a single model, underscoring the continued need for human oversight during theme development.	[24,30-32]
Quote extraction	Illustrative excerpts	Hallucination checks	Across the 5 studies evaluating primarily ChatGPT variants and a local Mistral-7B model, GAI-generated outputs included usable illustrative quotes. However, 4 of 5 studies reported fabricated, paraphrased, or inaccurately attributed quotes requiring manual correction. All 5 studies emphasized expert oversight and recommended hybrid human-AI workflows for quote verification and reporting.	[24,30,33-35]
Interpretation	Theory linking and implications	Reflexive sense making	Across 4 studies evaluating ChatGPT variants and a local Mistral-7B model, GAI performance declined when analyses required cultural, emotional, or theoretical interpretation. All 4 studies reported weaker theoretical grounding, reduced contextual insight, or difficulty with nuanced interpretation compared with human researchers. Although the severity of these limitations varied by model, no evaluated model consistently matched human performance in theory-driven interpretive analysis.	[8,24,26,36]

a ChatGPT variants were the most frequently evaluated systems across studies included in this synthesis. Evidence for Copilot, Bard-Gemini, Claude, Mistral, Llama, DeepSeek, and other models was more limited. Consequently, findings summarized at the level of “GAI” should not be interpreted as evidence that all systems perform similarly. where available, model-specific findings and differences are noted.

b LLM: large language model.

### Integration of GAI Across Qualitative Research Stages

During data familiarization, GAI tools were commonly used to summarize large volumes of text and identify surface-level patterns. Across studies, AI-generated summaries aligned broadly with manual familiarization; however, they consistently struggled with irony, emotional tone, and contextual nuance, particularly in culturally embedded data. As presented in Table 2, researchers emphasized the importance of human contextual verification at this early stage to prevent misinterpretation. For inductive coding, GAI tools were frequently applied as first-pass coders. Most studies reported strong concordance between AI-generated and human-generated codes, particularly for manifest content and frequently occurring concepts. Inductive codes produced by GAI tools were generally considered reliable and useful for accelerating early analytic phases; however, all studies underscored the need for continued human refinement and validation. In contrast, deductive coding revealed clearer boundaries to GAI performance.

While GAI tools achieved high accuracy for binary or concrete codes, performance declined for behavioral, interpersonal, and theoretically complex constructs. False positives and overapplication of codebook categories were common, reinforcing the value of hybrid workflows in which GAI tools accelerate code application, but humans correct errors to ensure conceptual fidelity. During theme development and interpretation, GAI tools were effective at clustering codes and identifying broad thematic structures but showed inconsistent ability to differentiate valence, capture latent meanings, or apply theory appropriately. Several studies reported that AI-generated themes resembled those produced by experts at a surface level; however, they lacked reflexive depth or introduced forced theoretical connections. These findings highlight that while GAI tools can support the initial stages of analysis, theoretical interpretation remains fundamentally human driven. GAI tools were often used for quote extraction; however, hallucinated or paraphrased quotes persisted even in studies using verification workflows.

### Reliability and Evaluation of GAI Relative to Human Analysis

Across the included studies, reliability and performance were commonly assessed by comparing GAI-generated codes, categories, themes, or summaries with those produced by human researchers. Measures of agreement included percent agreement, Cohen κ, Krippendorff α, Fleiss κ, Jaccard similarity, and semantic similarity measures. Evaluation also incorporated coding density, consistency, accuracy, sensitivity, specificity, precision, recall, *F*_1_-score, and overall accuracy, as well as qualitative assessments of thematic consistency, content overlap, and expert review (Table 3 and Appendix IV in Multimedia Appendix 4). Time efficiency was also frequently evaluated, with several studies reporting substantial reductions in analytic time relative to human-led approaches. Despite strong performance on many descriptive and structured coding tasks, human oversight remained necessary to verify outputs, identify hallucinations, assess interpretive depth, and ensure methodological rigor.

**Table 3.** Performance of generative AI (GAI) relative to human qualitative analysis^a^.

Analytic task	Typical agreement with human	Common metrics used	Noted limitations	References
Inductive thematic analysis	Across evaluated models (primarily ChatGPT variants, with additional evidence from Claude, DeepSeek, Gemma, and Llama), agreement with human thematic analysis was generally high, reaching approximately 80% in 1 study. Several models reliably identified core themes and extracted meaningful insights from large health-related datasets. Some studies reported that GAI-generated subthemes added complementary depth and that hallucinations were infrequent, whereas accuracy and comprehensiveness declined with longer texts and nuanced or divergent themes. Overall, evaluated models demonstrated value for theme identification, but inductive thematic analysis still requires human initiation, guidance, and oversight.	Coding quality (density, accuracy, and consistency), agreement and overlap (theme or subtheme alignment, conceptual overlap, and hallucinations), efficiency (analysis time), and evaluation scores, quantitative ratings across 6 quality dimensions (1-7) plus qualitative assessor feedback.	Human oversight was recommended in 5 of 6 studies. Limited depth, contextual understanding, or latent interpretation was reported in 4 of 6 studies. Performance depended on prompting strategies in 3 of 6 studies (50%), while variability across models or repeated runs was noted in 2 of 6 studies. Additional limitations included misclassifications or overinterpretation (2/6), reduced performance with longer texts (1/6), dependence on predefined themes (1/6), and limited theory-driven insight. Overall, although several ChatGPT, Claude, DeepSeek, Gemma, and Llama models demonstrated useful inductive thematic analysis capabilities; however, none consistently matched the depth and contextual richness of expert human analysis.	[23,26,37-40]
Deductive thematic analysis	Across evaluated models (primarily ChatGPT-3.5, ChatGPT-4, Bard, and Mixtral), GAI-generated themes were generally coherent, theory-aligned, and broadly consistent with human analyses, while substantially reducing analysis time. Agreement with human themes was higher for inductive analysis (~71% in 1 study) than deductive analysis (50%‐58% in 1 study), with overall human-AI coding agreement rated fair to moderate. Although performance varied across models and analytic approaches, ChatGPT-4, Bard, and Mixtral demonstrated potential as efficient complements to human-led qualitative analysis, particularly when integrated into hybrid workflows with researcher oversight.	Theme alignment and coherence with human codes, theoretical fit, theme consistency (% matched), intercoder agreement, and time efficiency; plus code frequencies, participant coverage, cross-checks (AI vs human), correctness flags, and similarity or readability scores (eg, ROUGE^b^/BERTScore^c^ and Flesch-Kincaid).	Human oversight or hybrid human-AI workflows were recommended in 3 of 4 studies, primarily to address limitations in contextual interpretation and ensure data integrity. Concerns regarding bias, hallucinations, unsupported findings, or privacy risks were also reported in 3 of 4 studies. Reduced depth, contextual understanding, or nuanced interpretation was also identified in 3 of 4 studies. Performance differences between evaluated models were noted across studies, suggesting that limitations were not uniform across all LLMs^d^. Lower coding reliability and coding organization limitations were reported less frequently (1/4).	[7,27,41,42]
Reflexive thematic analysis	Across the evaluated models (ChatGPT-3.5 and Mistral-7B), GAI-generated themes were broadly similar to those generated by experienced researchers and were capable of generating usable codebooks and preliminary thematic structures. ChatGPT-3.5 generated plausible thematic categories and theoretical interpretations, whereas Mistral-7B was more limited to surface-level summaries and keyword-based coding. Overall, tightly controlled prompting improved performance, but both models remained less interpretive and reflexive than expert human analysis.	Content coverage of key topics, accuracy of quotes and citations (including detection of fabricated quotes), and correctness of theoretical alignment. No numerical reliability metrics were reported.	Human oversight and critical verification were recommended in both studies. Both studies reported fabricated quotes and theory misapplication or forced theoretical interpretations, although these issues varied in severity across the evaluated models. One study identified surface-level coding, missed irony, language-sensitivity issues, and increased analytic workload due to additional verification requirements. Overall, neither ChatGPT-3.5 nor Mistral-7B replaced the reflexive, interpretive role of human researchers, despite providing useful support for triangulation and preliminary analysis.	[24,30]
Inductive content analysis	Performance varied, ChatGPT-3.5 and ChatGPT-4 generally provided accurate, CDC^e^-aligned HIV and PrEP^f^ information and, in 1 study, offered more culturally tailored and resource-specific guidance when race was specified. ChatGPT-4 conducted qualitative analysis much faster than novice human coders while producing a comparable volume of codes, categories, and themes, although human coders generated richer and more transparent analyses. Among health information tools, Bard-Gemini produced the most comprehensive but most variable responses, ChatGPT-4 generated the most consistent outputs, and the HIV.gov chatbot provided shorter but more citation-dense responses.	Comparison of answer differences by attitude and identity, counts of codes, categories, and themes, total coding time, and expert-assessed qualitative criteria (depth, contextual richness, and transparency).	Human oversight, expert review, or ongoing monitoring was recommended in all studies (4/4). Concerns regarding bias (3/4), transparency, explainability, or citation practices (3/4), and limited depth or contextual richness (2/4) were frequently reported. Hallucinated, fabricated, or inaccurate outputs were noted in 2 of 4 studies. One study found that outputs varied according to perceived user identity, creating opportunities for tailored messaging but also risks of unintended bias, while another (1/4) reported blurred distinctions between codes, categories, and themes. Overall, limitations differed across evaluated models, with ChatGPT, Bard-Gemini, and the HIV.gov chatbot exhibiting distinct strengths and weaknesses.	[30,43-45]
Deductive content analysis	Across evaluated systems (ChatGPT-3.5, ChatGPT-4, Copilot, and Llama 3.1), GAI-assisted coding generally showed moderate to high agreement with human coders (κ≈0.7‐0.96). ChatGPT closely replicated human annotations in structured tasks such as adverse-event detection, while Llama 3.1 achieved reliability comparable to human coding in large-scale content analysis. Copilot accurately reproduced manifest content but was weaker on latent interpretation, sometimes overinterpreting when additional contextual information was provided. Overall, performance was strongest for structured, deductive coding tasks, although meaningful differences were observed across models and analytic applications.	Precision of extracted mechanisms; intercoder reliability (Fleiss κ and Krippendorff α, including prevalence-adjusted κ); sensitivity (positive identification rate); reliability on full versus held-out samples; and qualitative agreement measures, completeness of meaning units, coding accuracy, similarity of sub- and over-arching themes, consensus with manual analysis, and output length or detail.	Human oversight or collaborative review was recommended in 3 of 6 studies. Reduced performance on complex, latent, or interpretive constructs was reported in 3 of 6 studies, particularly for Copilot and Llama when addressing nuanced or higher-order concepts. Bias, training data, or generalizability concerns were identified in 4 of 6 studies, spanning ChatGPT and Llama. Hallucinations, inaccuracies, or miscoding were reported in 2 of 6 studies, as were privacy and ethical concerns. Transparency and broader validation concerns were noted in 1 of 6 studies. Overall limitations varied across GAIs, suggesting that deficiencies were not uniform across all models despite generally strong performance on structured, manifest content coding tasks.	[36,38,46-49]
Grounded theory	Across the 2 studies of ChatGPT-4 and ChatGPT-4 Turbo, GAI-generated themes were broadly comparable to those produced by expert researchers and improved coding efficiency. Agreement was strongest for frequent, descriptive themes (>80% in 1 study), whereas performance declined for culturally or emotionally nuanced themes requiring deeper interpretation (~30% agreement in 1 study). Although overall theoretical frameworks were generally similar to those produced through manual coding, limitations in depth, contextual relevance, and coding organization remained evident. Overall, ChatGPT-4 usefully supports grounded theory coding but cannot replace human interpretive expertise.	Percent agreement, Cohen κ, node and reference counts, coverage rates, *t* tests, descriptive theme agreement percentages, and theme frequency counts.	Human oversight was recommended in both ChatGPT studies. Both studies reported limitations in depth, contextual understanding, and interpretive richness, as well as concerns regarding potential bias. One study identified difficulty with culturally grounded interpretation, while another reported hallucination and data privacy risks. Overall, the available evidence suggests that ChatGPT is useful for identifying surface-level themes and improving coding efficiency but cannot replace human expertise for nuanced theoretical and culturally embedded interpretation.	[8,41]

a ChatGPT (including GPT-3.5, GPT-4, GPT-4o, and GPT-4-Turbo) was the most frequently evaluated GAI system. Evidence for Copilot, Bard-Gemini, Claude, Llama, DeepSeek, Mistral, and other models was more limited. Consequently, findings summarized as “GAIs” reflect the available evidence base and should not be interpreted as evidence that all GAI systems perform similarly across qualitative analytic tasks.

b ROUGE: Recall-Oriented Understudy for Gisting Evaluation.

c BERTscore: Bidirectional Encoder Representations from Transformers Score.

d LLM: large language model.

e CDC: Centers for Disease Control.

f PrEP: pre-exposure prophylaxis.

### Performance of GAI Relative to Human-Led Qualitative Analysis

Across methodological approaches, GAI tools demonstrated their strongest performance in inductive thematic and content analysis, where agreement with human analysts commonly approached or exceeded 80% for core themes (Table 3). GAI tools reliably identified dominant patterns and accelerated analysis timelines, often completing tasks in a fraction of the time required for human-only workflows. However, performance consistently declined with longer texts, culturally nuanced data, or analyses requiring interpretation.

For deductive and reflexive thematic analysis, GAI tools produced coherent outputs aligned with predefined frameworks but struggled with nuance, reflexivity, and interpretive depth. Agreement with human coding was lower than with inductive approaches, and researchers frequently reported supporting superficial or keyword-driven outputs, reinforcing the point that reflexive qualitative methodologies require human interpretation and analysis. In grounded theory, GAI tools showed strong agreement on descriptive coding and frequent categories but were substantively weaker on culturally embedded or emotionally complex themes. While GAI tools improved efficiency and supported triangulation, they did not consistently generate theoretically robust core categories or relational structures, emphasizing that theory building remains a human-led intellectual task (Table 3). Across these 3 most common qualitative approaches (thematic analysis, content analysis, and grounded theory), performance variability was influenced by prompting strategies, model version, and the presence of predefined frameworks. Human oversight was universally identified as essential for ensuring interpretive accuracy, methodological rigor, and ethical compliance.

### General Findings Across Multiple Qualitative Approaches and GAI Applications

#### Overview

There were many qualitative approaches (eg, thematic analysis, content analysis, grounded theory, constant comparison, and framework analysis) used across the included studies, as well as many GAI models (eg, ChatGPT, Gemini, Copilot, and Claude). Below are general observations across all qualitative approaches, as well as differences across GAI models.

#### Inductive Thematic Analysis

Across studies using inductive thematic analysis, findings were both consistent and divergent. Some evidence shows limitations: ChatGPT-4 and OpenAI o1-preview underperformed on accuracy and comprehensiveness as document length increased, likely due to reasoning constraints [37]. In contrast, other research found strong performance. ChatGPT-4 achieved 85% expert-verified accuracy and high semantic alignment (0.795) when coding maternity care interviews, reducing coding time by 81% [28]. ChatGPT-4o also reproduced human themes from large free-text datasets, with keywords matching NVivo outputs, although human reviewers identified errors such as fabricated quotes, paraphrasing, and merged themes, underscoring the need for careful validation, particularly for latent meanings [33]. Older models, such as ChatGPT-3.5, still achieved 80% agreement with human coders, although they occasionally misclassified divergent subthemes due to overgeneralization or overfitting, reinforcing the need for human oversight [23].

Comparative studies show similar patterns. For inductive classification of social media data, ChatGPT-4o with 2-shot prompting outperformed DeepSeek V3, Gemma 3, Llama 3, and ChatGPT-3.5 across all metrics, although researchers emphasized the need for human-generated initial themes to guide GAI integration [39]. Another study comparing human and GAI analysis of interviews on asthma-related medication management needs found that Gemini, Copilot, and ChatGPT produced highly overlapping concepts with human NVivo coding, identifying the same 4 support domains but also misinterpreted nuances and generated themes absent from human analysis [34]. Similarly, Deiner et al [40] reported that ChatGPT-4 and Claude-2 consistently produced reasonable, relevant themes with low hallucination rates and strong capacity to process large social media datasets, although they still lacked the depth and consistency of expert analysts. Together, these studies suggest that GAI tools can substantially accelerate inductive thematic analysis but require rigorous human validation to ensure accuracy and interpretive depth.

#### Deductive and Reflexive Thematic Analysis

Several studies used deductive or reflexive thematic analysis when integrating GAI tools. A locally hosted Llama-2-70B-Instruct model applying Braun and Clarke’s 6-step reflexive thematic analysis demonstrated moderate-to-substantial similarity to human coding, suggesting that open-source models can support rigorous, secure, and scalable qualitative research [50]. In contrast, Vikan et al [24] found that another locally hosted model, Mistral-7B, produced only surface-level summaries, generated keyword-like codes, and failed to construct abstract or interpretive themes. The model also missed irony, fabricated quotes, introduced irrelevant theoretical links, and was highly sensitive to translation, thereby increasing researchers’ workload due to the required verification [24]. The authors concluded that high-quality thematic analysis remains fundamentally human driven.

Studies using off-the-shelf models found similar patterns. ChatGPT-4 outperformed ChatGPT-3.5 and Llama-3 when summarizing complex qualitative data from an online brain tumor support forum, producing efficient and consistent summaries [29]. ChatGPT-3.5-generated themes were broadly similar to those of an expert researcher in another study, although with fabricated quotes and forced theoretical interpretations, underscoring the need for human validation [30]. A study that applied ChatGPT-4 to analyze LGBTQ+ patients’ positive primary care experiences showed that the model accelerated theme development, but human oversight remained essential to ensure contextual accuracy and data integrity. This hybrid workflow appears promising for improving patient-provider research applications [42].

#### Mixed Inductive and Deductive Thematic Analysis Approaches

Several studies used mixed inductive-deductive thematic approaches. Sakaguchi et al [8] applied grounded theory and framework analysis to Japanese interviews using ChatGPT-4 and found more than 80% agreement with human coders for descriptive themes, but only approximately 30% agreement for culturally nuanced themes. The model was efficient for surface-level patterns but could not replace human interpretation [8]. Similarly, Kon et al [51] reported that ChatGPT-3.5 and ChatGPT-4 achieved 83% concordance with human analysis of community eye clinic interviews. Both models were approximately 20 times faster, with ChatGPT-4 producing fewer irrelevant subthemes [51]. ChatGPT-4 also scored higher than humans on confirmability, credibility, dependability, and consistency, although humans outperformed on transferability and depth [26]. In another study, ChatGPT-3.5 and Google Bard showed 71% inductive theme overlap but lower deductive consistency (50%‐58%) and only fair-to-moderate intercoder reliability; however, both completed analyses 97% faster than humans [7]. Finally, a study comparing a 2-step prompting workflow using ChatGPT-3.5-turbo with a local Mixtral 7×8B model found that both could generate coherent inductive and deductive themes aligned with human annotations and outperformed latent semantic analysis, demonstrating their usefulness as practical complements to qualitative workflows [27].

#### Content Analysis

Content analysis was the second most common qualitative method in this review. Several studies have demonstrated that LLMs can support aspects of content analysis, particularly in generating inductive, data-driven codes. For instance, ChatGPT-3.5-Turbo performed well in inductive coding and moderately well in deductive coding using frameworks such as the theoretical domains framework, with reliability improving through iterative refinement [48]. It also closely matched human annotators when detecting adverse events (AEs) across 10,000 Reddit posts, achieving more than 94% agreement for any AE and over 99% for serious AEs, suggesting strong potential for large-scale biomedical content analysis [49]. Yet generalizability and model variability remain concerns. Other studies found significant shortcomings: ChatGPT-3.5 and ChatGPT-4 produced superficial and sometimes inaccurate analyses when applied to the European Resuscitation Guidelines, including hallucinated content [47]. Similarly, ChatGPT-3.5 generated health information with more negative sentiment, higher reading levels, and lower DISCERN quality scores than Centers for Disease Control and Prevention (CDC) materials, underscoring the need for public literacy about AI-generated health information [36]. Studies evaluating smoking cessation guidance across multiple GPT-based models found only partially reliable outputs and inconsistent references to evidence-based treatments [52].

Comparisons across GAI systems showed varied performance. ChatGPT-3.5, ChatGPT-4, Google Bard, and the HIV.gov chatbot were all capable of providing accurate HIV medication safety information, although Bard was the most comprehensive and ChatGPT-4 the most consistent; the HIV.gov chatbot produced shorter but better-cited responses [44]. A smaller set of studies explored manifest and latent content analysis. Copilot reproduced manifest content reasonably well but produced shorter, less nuanced interpretations, performing best when paired with a structured framework such as Graneheim and Lundman’s [38]. In another study, ChatGPT-4 rapidly completed inductive content analysis and produced a comparable number of codes and categories, but human coders developed richer, more contextually grounded themes and maintained transparent audit trails [43]. Finally, a locally run Llama 3.1 model, used in an agentic workflow, effectively replicated and scaled a prior human-led content analysis, including multilingual data, although performance dropped for complex constructs such as efficacy; iterative prompting improved reliability [46].

#### Grounded Theory

Yue et al [41] provided guidance for using ChatGPT in grounded theory and compared ChatGPT-4-Turbo’s open, axial, and selective coding with human and software-assisted coding (NVivo). ChatGPT-4-Turbo produced reliable categories and theoretical structures and dramatically reduced analysis time (approximately 1 d for ChatGPT vs 3 wk for manual coding), although its coding lacked depth, contextual nuance, and strong relational connections [41]. Despite slight differences in core categories, ChatGPT-4-Turbo and manual approaches yielded largely similar theoretical frameworks, highlighting both the efficiency and limits of AI-assisted grounded theory [41]. Other studies used grounded theory as well, but as guidance for other qualitative methods, such as thematic analysis [8,30].

#### Thematic Narrative Analysis

Using thematic narrative analysis, Chubb et al [53] used ChatGPT-4o to extract themes and generate first-person vignettes from interview transcripts, substantially accelerating the synthesis while preserving participants’ voices. AI-generated themes uncovered patterns that sometimes differed from manual coding, prompting richer interpretation when triangulated with human review [53]. Overall, combining human expertise with ChatGPT-4o improved efficiency without compromising ethical standards or data fidelity [53].

#### Causal Loop Diagrams

ChatGPT-4 was used to replicate a 2019 causal loop diagram of an obesity prevention intervention by extracting variables, identifying causal links, and generating feedback loops [54]. It produced a richer set of feedback loops and captured new employee-level dynamics but occasionally exhibited directional errors and limited contextual nuance [54]. The authors of the study concluded that GAI is a useful complement to the research but not a replacement for human qualitative analysis [54].

#### Qualitative Description

Li et al [55] used an LLM called Versa (a private version of ChatGPT-4 that operates independently and stores no input data), in collaboration with human researchers, to analyze interviews on urology-related topics using a qualitative description approach. Versa consistently identified major themes with moderate agreement to human coding, but missed some nuanced subthemes, demonstrating limited depth compared to humans [55]. While human analysis remained contextually richer, the model’s reliability supports its role as a complementary tool in qualitative research [55].

#### Constant Comparison or Deductive Coding

Balt et al [22] tested whether the Llama-3 (70B instruct version) could reliably perform deductive coding and summarization of psychosocial-autopsy interview data. The model achieved 84% accuracy on binary coding and produced adequate or good summaries in approximately 80% of cases; in 1.3% of cases, the summarized fragments were hallucinated or miscoded content [22]. It processed 38 interviews in 8 days, compared with 4 months for the human-led analysis; however, the authors recommend a collaborative workflow in which the LLM performs initial coding, followed by expert review and refinement [22]. Another study had similar conclusions, finding that LLM-assisted (ChatGPT-4o and Gemini Advanced Pro 1.5) thematic analysis with the constant comparison method quickly identified recurring patterns but often missed emotional nuance and contextual depth [35]. The human-led analysis captured more complex experiences; it required much more time [35]. Overall, the authors recommend a hybrid approach (human and LLM) for this type of qualitative methodology.

#### Query-Based Analysis, Autoethnographic Case Study, and Qualitative Case Study

Morgan [32] introduced a 3-step query-based analysis (QBA) workflow using ChatGPT-3.5 and ChatDOC to generate themes, subthemes, and illustrative quotes from qualitative health data. QBA produced 5 themes and subthemes and efficiently streamlined steps 3 to 5 of Braun and Clarke’s framework while maintaining interpretive depth, although further validation is still needed. Similarly, Ferguson [31] used ChatGPT-3.5 in an autoethnographic case study of comments on a newspaper article and found that the model failed to capture sentiment distinctions in satirical posts, highlighting the importance of clear prompting. To evaluate prompting strategies, Nair et al [25] compared Flan-T5, ChatGPT-3, and ChatGPT-3.5 for summarizing patient forum posts. Zero-shot prompting performed reasonably well, but ChatGPT-3.5 with directional-stimulus prompting in a 3-shot setup achieved the strongest ROUGE (Recall-Oriented Understudy for Gisting Evaluation) and BERTScore (Bidirectional Encoder Representations from Transformers Score) performance, producing accurate, plausible summaries [25]. The findings suggest that pretrained LLMs can generate meaningful summaries that enhance understanding of patient needs, although the study was limited by a small dataset, reliance on a single human annotator, and uncertain generalizability across models or prompting strategies [25].

#### Qualitative Comparative Analysis (QBA), Expert Appraisal

Three GAI tools (ChatGPT-4o, Gemini 1.5 Pro, and Grok) were tested for their ability to interpret and refine medical definitions of clinical obesity using structured prompts across baseline, contextual, and generative rounds [56]. When provided with authoritative context (eg, the Lancet Commission Report), models produced more precise, clinically aligned definitions, suggesting GAI tools can support knowledge synthesis when paired with expert oversight [56]. Bragazzi and Garbarin [57] similarly found that ChatGPT-4 correctly labeled 85% of sleep-related myths as false and aligned well with expert ratings, although explanations were more general than expert technical responses. Conversely, a study evaluating ChatGPT-4o, Perplexity, Llama-3 70B, and a Retrieval-Augmented Generation–enhanced Llama-3-70B model across 30 guideline-based cardiovascular disease nutrition questions showed that the Retrieval-Augmented Generation model provided the most reliable, guideline-adherent answers with no harmful content [58]. However, its outputs were less readable because they drew directly from technical guideline language [58].

### Ethical Considerations of the Application of GAI-Assisted Qualitative Research

Ethical considerations were inconsistently reported across studies but emerged as a critical cross-cutting theme. As summarized in Table 4, most studies mitigated privacy risks through data deidentification and, in some cases, using enterprise- or company-hosted or locally hosted models. However, privacy risks, especially when using third-party cloud services that could retain data for training, were highlighted, and AI-specific participant consent was rarely described.

**Table 4.** Ethical and governance approaches reported across studies^a^.

Ethical domain	Mitigation strategies	Gaps identified	References
Data privacy	Deidentification of data; enterprise (company-protected) versions of GAIs or use of local models.	Cloud retention policies unclear.	[7,27,28,33,38,40,46,53-55,59]
Consent and IRB^b^ review	IRB approval or exemption reported.	AI-specific consent rarely described.	[30,33,37,45,50-52,58,60]
Hallucination risk	Human verification workflows.	Errors still detected, the challenge of AI being a “black box” remains.	[7,22,24,25,28,35,41,42,46,54,56,57,61]
Bias and equity	Prompt refinement; reflexive review.	Algorithmic bias seldom evaluated.	[24,36,40,45,50,55]
Transparency	Disclosure of GAI use.	Inconsistent reporting of prompts used or version of GAI being used (paid vs free).	[24,25,35,39,53,54]

a Mitigation strategies reflect practices reported by study authors and are not specific to any single generative AI (GAI) model.

b IRB: institutional review board.

Authors stressed the importance of deidentifying data and obtaining institutional review board (IRB) or ethical approval before inputting participant-generated text into a GAI. Many studies were exempt from IRB review because the data were anonymized or publicly available. Hallucinations, fabricated quotes, and misinformation were common, prompting calls for human validation, post-processing, and clear researcher responsibility. Bias and equity were frequently evaluated explicitly, despite evidence that outputs varied by perceived user identity or prompting strategies used. Transparency practices, such as reporting model version, access type, and prompting strategies, were inconsistent, limiting reproducibility and comparability across studies (Table 4).

### Trade-Offs in Using GAI for Qualitative Health Research

Across studies, the use of GAI in qualitative health research was consistently characterized by identifiable trade-offs between efficiency, reliability, interpretive depth, and ethical considerations. Table 5 synthesizes these trade-offs as explicitly reported or implied across the included studies.

**Table 5.** Trade-offs in using generative AI (GAI) for qualitative health research^a^.

Dimension	Advantages	Limitations
Efficiency	Dramatically reduces time for coding, summarizing, and scaling analysis (up to 97% less time compared to human coders).	Time savings partly offset by verification, correction, and preparation prior to running the GAI.
Reliability	Comparable to humans for manifest or descriptive coding.	Lower reliability for latent or culturally embedded analysis.
Interpretive depth	Useful for identifying surface patterns.	Lacks reflexivity, emotional insight, and contextual understanding.
Transparency	Outputs are reproducible if prompts are held constant; audit trails are important for transparency.	Models remain as “black boxes.”
Bias and hallucination	Detectable through human review.	Training data bias of the GAIs and fabricated quotes remain risks.
Ethics and privacy	Local or enterprise or company models can mitigate risks.	Commercial cloud tools pose confidentiality concerns.
Equity and access	Enables large-scale analysis with limited staff.	Subscription costs, computer or hardware high costs, negative environmental impact.
Best use case	First-pass analysis, identifying surface level or manifest content, can support triangulation. Should be used in hybrid human and AI approach across different qualitative health methodologies.	It should not replace human involvement or interpretation, particularly for analyzing latent content or reflexive analysis.

a Trade-offs represent an overall synthesis of the reviewed literature. Because most studies evaluated ChatGPT variants, findings may not apply equally to all GAI models.

In terms of efficiency, GAI tools dramatically reduced the time required for coding, summarization, and large-scale qualitative analysis, with several studies reporting analysis times up to 97% faster than human-led analysis. These time savings were most pronounced during first-pass coding, surface pattern identification, and processing large textual datasets. However, studies also noted that these gains were partially offset by the need for preparation (eg, prompt development and data cleaning) and post-processing tasks, including output verification, error correction, and detection of fabricated or hallucinated content. Regarding reliability, GAI tools generally performed comparably to human coders for manifest descriptive and frequently occurring content. Reliability decreased for analyses requiring latent interpretation, cultural sensitivity, or emotionally embedded meaning, with poorer performance on nuanced themes or culturally specific constructs, reinforcing that reliability varied systematically by analytic depth. Across studies, interpretive depth emerged as a key limitation. GAI tools were effective at identifying surface-level patterns and recurring topics but consistently struggled with reflexivity, emotional resonance, and context. While some AI-generated themes aligned structurally with human analyses, they often lacked the conceptual richness, reflexive richness, reflexive reasoning, and theoretical grounding present in human-led expert qualitative interpretation.

With respect to transparency, multiple studies noted that AI outputs were reproducible when prompts and inputs were held constant, and audit trails could be maintained through careful documentation. Similarly, authors highlighted the persistent opacity of model training and decision processes, limiting interpretability and raising concerns about methodological transparency. Bias and hallucination risk were reported across nearly all analytic stages. While fabricated quotes, paraphrasing errors, and biased framing were often detectable through human review, they remained recurrent risks even in studies using structured prompts and verification workflows. These findings underscore that bias and hallucination were not isolated anomalies but systematic concerns requiring ongoing oversight. Ethical and practical considerations related to privacy, equity, and access further shaped reported trade-offs. The use of local or enterprise-level models reduced confidentiality risks, whereas commercial cloud-based tools raised concerns about data retention and participant privacy. Access barriers, including subscription fees, computational requirements, and infrastructure demands, were noted as limiting the feasibility of GAI adoption for some researchers and institutions, and concerns about the environmental impact of GAI were also raised.

Taken together, the results summarized in Table 5 indicate that GAI tools offer substantial practical advantages for early-stage and large-scale qualitative analysis, while acknowledging limitations in the interpretive, reflexive, and ethically sensitive aspects of qualitative health research. These trade-offs were consistent across qualitative methodologies and GAI platforms in this review.

### CASP Appraisal of the Included Studies

Overall, the body of evidence appraised using the CASP checklist demonstrates strong methodological quality across the studies, particularly in relation to the research objectives, use of qualitative methodologies, appropriate research designs, clear data collection procedures, and the presentation of findings. Most studies addressed ethical considerations, often through IRB approval, data deidentification, or justification for exemption from IRB review, and rigor in data analysis using established qualitative frameworks (eg, Braun and Clarke, 2006; and Graneheim and Lundman, 2004), and in some cases, quantified reliability metrics (eg, Fleiss κ and Krippendorff α). However, several methodological limitations were identified across the studies, most notably incomplete articulation of theoretical or epistemological positioning and limited attention to reflexivity, with many studies either briefly acknowledging or omitting discussion of researcher positionality and its influence on analysis. Recruitment strategies and sampling rationales were appropriate, as many papers were methodological comparisons or exploratory; some relied on small convenience or secondary datasets, constraining transferability. Collectively, the CASP appraisal indicates that while the studies provide credible and valuable methodological insights, particularly regarding AI-assisted qualitative analysis, the literature consistently supports a hybrid human-AI approach, underscoring the continued necessity of human interpretive expertise for reflexive depth, contextual nuance, and theoretical alignment. See Appendix V in Checklist 2 for the full CASP checklist completed for each study.

## Discussion

A clear consensus across qualitative methodologies emphasizes that a hybrid approach combining GAI with human expertise is essential for maintaining credibility, reliability, rigor, and ethical standards in qualitative health research [7,35,42,59]. While GAI offers efficiency, scalability, and strong pattern recognition capabilities, it cannot replace the nuanced judgment, contextual insight, or reflexive decision-making of trained qualitative researchers. Used together, GAI tools can streamline data handling and preliminary coding, while humans provide interpretive depth, methodological grounding, and ethical oversight. This hybrid model strengthens validity and ensures analyses remain rooted in lived experiences and social contexts. Despite rapid adoption, the literature reveals significant inconsistency in methodological reporting. Many studies include qualitative components but do not clearly identify the qualitative methodology guiding the analysis [56-58]. As qualitative approaches differ in philosophy and purpose, it is essential to name both the method (eg, grounded theory, content analysis, and thematic analysis) and the analytic approach (eg, inductive, deductive, semantic, latent, and manifest). As GAI tools become more integrated into research workflows, transparent methodological specification is critical for evaluating whether AI use enhances or undermines rigor. Conceptual ambiguity also persists, with “LLM” and “GAI” often used interchangeably, although GAI tools represent only one category of LLMs. Clear definitions will reduce confusion and improve consistency in reporting.

The rapid evolution of GAI technologies also has implications for how evidence in this field is synthesized, updated, and translated into best practices. Consequently, the surge in publications over the past 2 years shows that GAI is entering qualitative health research faster than methodological guidance can keep pace. As traditional systematic reviews are slow and often outdated by the time they are published, rapid reviews may offer a more practical way to update best practices in a fast-moving technological environment [17]. Journals are increasingly receptive to rapid evidence synthesis, enabling more timely recommendations while maintaining transparent and systematic processes. Across studies, authors should report a standardized set of elements to support comparison, evaluation, and replication. Drawing on Steckler and McLeroy’s [62] external validity framework, these include recruitment and representativeness, consistency of implementation, impacts across outcomes, and information on attrition or sustainability. For GAI-specific contexts, additional reporting is needed: the model used, access mechanism (free interface, subscription, API, or local installation), version number, prompting strategy, and data handling procedures. Such disclosures improve comparability and highlight the practical constraints and opportunities associated with different GAI tools.

As GAI becomes more embedded in research practice, formal training will be essential. Integrating structured GAI instruction into public health education, especially Master of Public Health programs, would help future professionals understand both the capabilities and limits of these tools. Curricula could include modules on prompt engineering, GAI-supported qualitative and quantitative methods, and responsible use topics such as privacy, bias mitigation, reproducibility, and transparency. Faculty development is equally important, including short courses on GAI for research, ethics seminars, prompt engineering laboratories, and workshops on transparent AI-assisted writing. Together, these efforts would build a workforce skilled in using GAI while upholding ethical and scientific standards [14,63].

Beyond researcher training and technical competency, the reviewed studies also highlight the importance of governance frameworks for ensuring the responsible, transparent, and accountable use of GAI in qualitative health research. Across the included studies, governance concerns extended beyond technical performance and frequently encompassed privacy, transparency, accountability, bias, hallucinations, and ethical oversight. Several studies mitigated privacy risks through deidentification procedures, enterprise-protected platforms, or locally hosted models, yet concerns remained regarding cloud-based data retention, confidentiality, and the use of sensitive qualitative data in commercial models (eg, ChatGPT). These findings align with broader governance literature, which argues that GAI should be viewed as a sociotechnical system requiring oversight not only of the technology itself but also of the people, data, organizational processes, and societal contexts in which it is used. For example, Janssen [64] proposed a responsible governance framework based on a complex adaptive systems perspective, emphasizing public values, data provenance, joint accountability, risk assessment, communications, and continuous oversight as key components of responsible GAI deployment. The author further argues that governance should evolve alongside GAI technology and include multiple lines of defense rather than relying on a single safeguard [64].

The governance challenges identified in this review are also consistent with broader concerns raised by Taeihagh [65], who noted that GAI governance must address hallucinations, opacity, bias amplification, privacy violations, misinformation, data governance, and accountability gaps through adaptive, participatory, and proactive approaches. Taeihagh [65] further emphasized the importance of impact assessments, auditing, transparency requirements, stakeholder engagement, and governance mechanisms capable of responding to rapidly evolving technological capabilities. Emerging governance research in higher education demonstrates increasing institutional emphasis on approved tool lists, disclosure requirements, data protection policies, privacy safeguards, and risk management frameworks in the integration of GAI into organizational practice [65]. These approaches reflect a broader shift toward balancing innovation with accountability and regulatory compliance [65]. Taken together, the future integration of GAI into qualitative health research should be accompanied by formal governance structures that promote transparency, protect participant data, clarify accountability for AI-assisted outputs, and ensure that human researchers retain ultimate responsibility for interpretation, decision-making, and ethical conduct [65,66].

Equity concerns around access to GAI tools remain significant. Not all GAI tools are free, and many advanced models require paid subscriptions or API credits, creating cost barriers for researchers and institutions with limited resources. For example, ChatGPT’s more powerful models are available only via subscription or paid API access, with token costs that vary by model and often increase for newer versions. Some institutional GAI tools, such as enterprise versions of Copilot, offer enhanced data protections but only through paid plans, creating disparities in access to privacy-preserving features. Likewise, locally hosted or high-parameter models require substantial hardware capacity that is not universally available, further deepening inequities. Environmental inequities compound these challenges. The physical infrastructure supporting GAI, semiconductor manufacturing, data center operations, and large-scale model training consumes vast amounts of water, energy, and raw materials. The carbon and water footprints of model training are also substantial; training GPT-3 alone required an estimated 1287 MWh of electricity and generated more than 500 tons of CO₂ [67]. These environmental pressures highlight the importance of transparent reporting and thoughtful policy development to avoid reinforcing existing inequities [67].

Equity concerns emerged not only in access to GAI tools but also in the analytic outputs they produce. Several studies have shown that GAI tools tend to identify surface-level themes more reliably while underperforming on culturally embedded, linguistically nuanced, or emotionally complex data, raising concerns that analyses may be biased toward English-speaking populations [8,24,44]. For example, agreement between GAI and human-generated themes dropped substantially from 80% for descriptive themes to 30% for culturally (Japanese) nuanced themes [8]. Concerning health literacy, health information generated from GAI, specifically ChatGPT, required a higher reading grade level, while information from the CDC was of higher quality compared to the information provided by ChatGPT [36]. Importantly, only one study included in this review examined the algorithmic bias of GAI tools systematically. Chandler et al [45] examined whether ChatGPT provides HIV prevention and pre-exposure prophylaxis differs based on the user’s race and explored how this GAI could inform public health education for Black women self-educating about sexual health. ChatGPT consistently provided accurate, CDC-aligned information on HIV prevention and pre-exposure prophylaxis. When the user was described as Black, responses included more detailed, culturally attuned guidance and specific financial assistance resources, whereas generic prompts (no race specified) produced broader, less targeted overviews [45]. These variations show that ChatGPT’s outputs can shift based on perceived user identity, creating opportunities for more equitable, tailored messaging but also risks of unintended bias without careful monitoring [45]. Taken together, the evidence suggests that GAI-assisted qualitative analysis risks reinforcing health inequities by amplifying widely represented narratives and diminishing marginalized experiences, underscoring the necessity of reflexive, human-led equity evidence when GAI tools are used in health-related qualitative research.

On the basis of the included studies, several practical recommendations emerge for researchers seeking to integrate GAI into qualitative health research. First, GAI should be used within a hybrid human-AI workflow, with researchers retaining responsibility for interpretation, validation, and reflexive analysis. Second, GAI outputs, including codes, themes, summaries, and quotations, should be systematically verified for inaccuracies, hallucinations, and contextual misinterpretations. Third, researchers should transparently report the GAI model, version, prompting strategy, and data handling procedures to support reproducibility and methodological rigor. Fourth, sensitive data should be protected through deidentification procedures and, where feasible, the use of enterprise-protected or locally hosted models. Finally, GAI appears most appropriate for data management, summarization, and first-pass coding tasks, whereas latent, reflexive, theory-driven, and culturally nuanced analyses should remain primarily human led.

This rapid review is not without limitations; this type of review often streamlines key steps of full systematic reviews, which can introduce bias by limiting the search scope, relying on a single reviewer, and conducting a partial review by a second reviewer. As the search was restricted to 2022 to December 2025, some recently published studies may have been missed. Articles were included only if they were published in English, which could have excluded papers in other languages, and the systematic search was conducted only in 3 major databases. This rapid review intentionally excluded studies focused solely on clinical applications of GAI, such as medical documentation, clinical decision support, surgical applications, and medical education. Although this decision allowed the review to maintain a focused examination of GAI within qualitative health research methodologies, it may have excluded relevant evidence from the broader health care AI literature. A substantial and rapidly growing body of research already examines GAI-supported clinical workflows, and future reviews should synthesize this literature separately.

In addition, the included studies used a wide range of qualitative methodologies, including thematic analysis, content analysis, and grounded theory, limiting direct comparability across studies evaluating ChatGPT variants, whereas other GAI models such as Copilot, Bard-Gemini, Claude, Llama, DeepSeek, and Mistral were evaluated less frequently, potentially limiting the generalizability of findings across the broader landscape of GAI models. Furthermore, few studies examined the influence of researcher familiarity, prompt engineering expertise, or prior experience with specific GAI models. Variations in user knowledge and prompting practices may have affected the quality and performance of GAI outputs, yet these factors were rarely measured, compared, or reported, making it difficult to distinguish model-specific capabilities from differences in user expertise. However, despite these limitations, a rapid review remains an appropriate approach for assessing the current state of evidence on a rapidly evolving technology such as GAI.

The debate over GAI’s role in qualitative research remains active, especially in methodologies emphasizing deep reflexivity, such as reflexive thematic analysis [6]. While some scholars warn that GAI could dilute interpretive depth or produce fabricated content, others argue that its widespread availability makes it unrealistic to exclude it from research practices [6,9]. As with the earlier introduction of computer-assisted qualitative software, the field must adapt by developing approaches that harness GAI’s benefits while preserving the interpretive richness that defines qualitative inquiry [6,9]. Across the studies included in this rapid review, recurring risks, including algorithmic bias, hallucinations, erosion of interpretive nuance, and privacy breaches, can be mitigated through human-in-the-loop workflows, rigorous prompt engineering, transparent audit trails, validation against human coding, and careful use of deidentified or local data.

Importantly, this new age of GAI requires researchers to be explicit and strategic about what GAI is and what it is not used for. Appropriate uses may include data management tasks, such as transcription support, summarization, organization, and first-pass or descriptive coding, where efficiency gains do not substitute for interpretive judgment. GAI should be used with caution in inductive, latent, or theory-driven analyses and should never replace researcher-led efforts, particularly in reflexive, interpretive, or epistemologically generative phases of analysis. When GAI is used beyond descriptive phases or preparatory stages, outputs must be treated as provisional and systematically checked against the data, theory, and the researcher’s reflexive engagement. Thus, rather than automating qualitative interpretation, GAI should function as a tool whose contributions are documented, scrutinized, and subordinated to human interpretation. Maintaining an open, reflexive, and evolving dialogue will allow the field to develop shared norms, methodological guidance, and ethical guardrails for responsible, transparent, and equitable integration of GAI in qualitative health research. This review positions GAI not only as a methodological tool but as a force that challenges how qualitative knowledge is produced, interpreted, and validated in health research.

## Supplementary material

10.2196/98551 Multimedia Appendix 1 Boolean logic syntax using all keyword combinations.

10.2196/98551 Multimedia Appendix 2 Full search strategy.

10.2196/98551 Multimedia Appendix 3 Data extraction variables.

10.2196/98551 Multimedia Appendix 4 Main findings of generative AI application, reliability, evaluation metrics, and ethics.

10.2196/98551 Checklist 1 PRISMA checklist.

10.2196/98551 Checklist 2 CASP checklist.

## Abbreviations

AE: adverse event
BERTscore: Bidirectional Encoder Representations from Transformers Score
CASP: Critical Appraisal Skills Programme
CDC: Centers for Disease Control
GAI: generative AI
IRB: Institutional Review Board
JBI: Joanna Briggs Institute
LLM: large language model
PRISMA: Preferred Reporting Items for Systematic Reviews and Meta-Analyses
PROSPERO: International Prospective Register of Systematic Reviews
QBA: query-based analysis
ROUGE: Recall-Oriented Understudy for Gisting Evaluation
SPIDER: sample, phenomenon of interest, design, evaluation, research type

## References

[1] Wheldon C, McKee R (2025). AI-empowered qualitative data analysis. CH.

[2] Stickley T, O’Caithain A, Homer C (2022). The value of qualitative methods to public health research, policy and practice. Perspect Public Health.

[3] Owoahene Acheampong I, Nyaaba M Review of qualitative research in the era of generative artificial intelligence. SSRN.

[4] Zhang H, Wu C, Xie J, Lyu Y, Cai J, Carroll JM (2025). Harnessing the power of AI in qualitative research: exploring, using and redesigning ChatGPT. Computers in Human Behavior: Artificial Humans.

[5] Banh L, Strobel G (2023). Generative artificial intelligence. Electron Markets.

[6] Jowsey T, Braun V, Clarke V, Lupton D, Fine M (2025). We reject the use of generative artificial intelligence for reflexive qualitative research. Qualitative Inquiry.

[7] Prescott MR, Yeager S, Ham L (2024). Comparing the efficacy and efficiency of human and generative AI: qualitative thematic analyses. JMIR AI.

[8] Sakaguchi K, Sakama R, Watari T (2025). Evaluating ChatGPT in qualitative thematic analysis with human researchers in the Japanese clinical context and its cultural interpretation challenges: comparative qualitative study. J Med Internet Res.

[9] Monforte J (2026). Generative artificial intelligence and the craft of qualitative health research: observations from a techno-negative stance. Qual Health Res.

[10] Kosmyna N, Hauptmann E, Yuan YT, Situ J, Liao XH, Beresnitzky AV (2025). Your brain on ChatGPT: accumulation of cognitive debt when using an AI assistant for essay writing task. arXiv.

[11] Gerlich M (2025). AI tools in society: impacts on cognitive offloading and the future of critical thinking. Societies.

[12] Dellepiane P Artificial. La nueva inteligencia y el contorno de lo humano. TEyET.

[13] Sigman M, Bilinkis S (2023). Artificial: La Nueva Inteligencia y El Contorno de Lo Humano.

[14] Davison RM, Chughtai H, Nielsen P (2024). The ethics of using generative AI for qualitative data analysis. Information Systems Journal.

[15] Brereton E (2025). Colleges and universities offer faculty development for AI use in the classroom. EdTech Magazine.

[16] Page MJ, McKenzie JE, Bossuyt PM (2021). The PRISMA 2020 statement: an updated guideline for reporting systematic reviews. BMJ.

[17] Tricco AC, Khalil H, Holly C (2022). Rapid reviews and the methodological rigor of evidence synthesis: a JBI position statement. JBI Evid Synth.

[18] Cooke A, Smith D, Booth A (2012). Beyond PICO: the SPIDER tool for qualitative evidence synthesis. Qual Health Res.

[19] Ouzzani M, Hammady H, Fedorowicz Z, Elmagarmid A (2016). Rayyan-a web and mobile app for systematic reviews. Syst Rev.

[20] CASP Qualitative Studies Checklist. CASP.

[21] Long HA, French DP, Brooks JM (2020). Optimising the value of the critical appraisal skills programme (CASP) tool for quality appraisal in qualitative evidence synthesis. Research Methods in Medicine & Health Sciences.

[22] Balt E, Salmi S, Bhulai S (2025). Deductively coding psychosocial autopsy interview data using a few-shot learning large language model. Front Public Health.

[23] Castellanos A, Jiang H, Gomes P, Vander Meer D, Castillo A (2025). Large language models for thematic summarization in qualitative health care research: comparative analysis of model and human performance. JMIR AI.

[24] Vikan M, Aryan R, Kannelønning MS, Riegler MA, Danielsen SO (2026). Reflecting on LLM support in reflexive thematic analysis: an exploratory study. Qual Health Res.

[25] Nair RAS, Hartung M, Heinisch P (2025). Summarizing online patient conversations using generative language models: experimental and comparative study. JMIR Med Inform.

[26] Kondo T, Miyachi J, Jönsson A, Nishigori H (2024). A mixed-methods study comparing human-led and ChatGPT-driven qualitative analysis in medical education research. Nagoya J Med Sci.

[27] Wosny M, Hastings J (2024). Applying large language models to interpret qualitative interviews in healthcare. Stud Health Technol Inform.

[28] Qiao S, Fang X, Wang J, Zhang R, Li X, Kang Y (2025). Generative AI for thematic analysis in a maternal health study: coding semistructured interviews using large language models. Appl Psychol Health Well Being.

[29] Muasher-Kerwin C, Hughes MC, Foster ML, Al Azher I, Alhoori H (2025). Exploring large language models for summarizing and interpreting an online brain tumor support forum. Digit Health.

[30] Wachinger J, Bärnighausen K, Schäfer LN, Scott K, McMahon SA (2025). Prompts, pearls, imperfections: comparing ChatGPT and a human researcher in qualitative data analysis. Qual Health Res.

[31] Ferguson C (2025). A researcher’s journey to the use of AI for qualitative data analysis: findings from a test case with ChatGPT. Issues Educ Res.

[32] Morgan DL (2026). Query-based analysis: a strategy for analyzing qualitative data using ChatGPT. Qual Health Res.

[33] Goldberg EM, Macis M, Bounds M, Picazo JG, Nicholas LH (2025). Free-text responses in a nationally representative experimental survey about end-of-life care choices: ChatGPT-4o-assisted qualitative analytical study. JMIR Aging.

[34] Jeminiwa RN, Popielaski C, King A (2025). Exploring young adults’ experiences and beliefs in asthma medication management: pilot qualitative study comparing human and multiple AI thematic analysis. JMIR Form Res.

[35] Shanwetter Levit N, Saban M (2025). When investigator meets large language models: a qualitative analysis of cancer patient decision-making journeys. NPJ Digit Med.

[36] Young A, Omosun F (2025). A comparative analysis of CDC and AI-generated health information using computer-aided text analysis. J Commun Healthc.

[37] Han Z, Tavasi A, Lee J (2025). Can large language models be used to code text for thematic analysis? An explorative study. Discov Artif Intell.

[38] Lund-Tonnesen M, Vahr Lauridsen S, Rosenberg J (2025). Evaluating Microsoft Copilot in qualitative health research: accurate for manifest content coding but limited in latent interpretation. Cureus.

[39] Hairston JM, Ranjan R, Lakamana S (2025). Automating inductive thematic analyses of health content using large language models: a proof-of-concept study using social media data. JAMIA Open.

[40] Deiner MS, Honcharov V, Li J, Mackey TK, Porco TC, Sarkar U (2024). Large language models can enable inductive thematic analysis of a social media corpus in a single prompt: human validation study. JMIR Infodemiology.

[41] Yue Y, Liu D, Lv Y, Hao J, Cui P (2025). A practical guide and assessment on using ChatGPT to conduct grounded theory: tutorial. J Med Internet Res.

[42] Stage MA, Creamer MM, Ruben MA (2025). “Having providers who are trained and have empathy is life-saving”: improving primary care communication through thematic analysis with ChatGPT and human expertise. PEC Innov.

[43] Lockwood A, Newman DS, Mossing KW, Glubzinski A, Cohen E (2026). Human versus machine: a comparative analysis of qualitative coding by humans and ChatGPT-4. Sch Psychol.

[44] Beegle S, Gomez LA, Blackard JT (2025). HIV prevention and treatment information from four artificial intelligence platforms: a thematic analysis. AIDS Behav.

[45] Chandler RD, Warner S, Aidoo-Frimpong G, Wells J (2024). “What did you say, ChatGPT?” The use of AI in Black Women’s HIV self-education: an inductive qualitative data analysis. J Assoc Nurses AIDS Care.

[46] Farjam M, Meyer H, Lohkamp M (2026). A practical guide and case study on how to instruct LLMs for automated coding during content analysis. Soc Sci Comput Rev.

[47] Beck S, Kuhner M, Haar M, Daubmann A, Semmann M, Kluge S (2024). Evaluating the accuracy and reliability of AI chatbots in disseminating the content of current resuscitation guidelines: a comparative analysis between the ERC 2021 guidelines and both ChatGPTs 3.5 and 4. Scand J Trauma Resusc Emerg Med.

[48] Bijker R, Merkouris SS, Dowling NA, Rodda SN (2024). ChatGPT for automated qualitative research: content analysis. J Med Internet Res.

[49] Leas EC, Ayers JW, Desai N, Dredze M, Hogarth M, Smith DM (2024). Using large language models to support content analysis: a case study of ChatGPT for adverse event detection. J Med Internet Res.

[50] Mathis WS, Zhao S, Pratt N, Weleff J, De Paoli S (2024). Inductive thematic analysis of healthcare qualitative interviews using open-source large language models: How does it compare to traditional methods?. Comput Methods Programs Biomed.

[51] Kon MHA, Pereira MJ, Molina JADC, Yip VCH, Abisheganaden JA, Yip W (2025). Unravelling ChatGPT’s potential in summarising qualitative in-depth interviews. Eye (Lond).

[52] Abroms LC, Yousefi A, Wysota CN, Wu TC, Broniatowski DA (2025). Assessing the adherence of ChatGPT chatbots to public health guidelines for smoking cessation: content analysis. J Med Internet Res.

[53] Chubb LA, Jackson S, Naseer B, Matthews M (2026). To leave or stay? Influences on early exit and completion in a New Zealand residential drug rehabilitation service. Qual Health Res.

[54] Jalali MS, Akhavan A (2024). Integrating AI language models in qualitative research: replicating interview data analysis with ChatGPT. Syst Dyn Rev.

[55] Li KD, Fernandez AM, Schwartz R (2024). Comparing GPT-4 and human researchers in health care data analysis: qualitative description study. J Med Internet Res.

[56] Kermansaravi M, Cohen RV (2025). Clinical obesity through the lens of context-aware large language models. Obes Surg.

[57] Bragazzi NL, Garbarino S (2024). Assessing the accuracy of generative conversational artificial intelligence in debunking sleep health myths: mixed methods comparative study with expert analysis. JMIR Form Res.

[58] Parameswaran V, Bernard J, Bernard A (2025). Evaluating large language models and retrieval-augmented generation enhancement for delivering guideline-adherent nutrition information for cardiovascular disease prevention: cross-sectional study. J Med Internet Res.

[59] Misgav K, Neufeld-Kroszynski G, Palombo M, Karnieli-Miller O (2026). Human analysis vs. artificial intelligence: analyzing of qualitative medical students’ narratives. Qual Health Res.

[60] Keating C, Marcus SC, Bowden CF, Worsley D, Doupnik SK (2025). Artificial intelligence and qualitative analysis of emergency department telemental health care implementation survey. Telemed J E Health.

[61] Liu X, He L, Alanazi E, Liu E, Goss A, Gumireddy L (2025). Assessing the accuracy and explainability of using ChatGPT to evaluate the quality of health news. BMC Public Health.

[62] Steckler A, McLeroy KR (2008). The importance of external validity. Am J Public Health.

[63] Marshall DT, Naff DB (2024). The ethics of using artificial intelligence in qualitative research. J Empir Res Hum Res Ethics.

[64] Janssen M (2025). Responsible governance of generative AI: conceptualizing GenAI as complex adaptive systems. Policy and Society.

[65] Taeihagh A (2025). Governance of generative AI. Policy and Society.

[66] LaFrance J (2026). Governing generative artificial intelligence: institutional policies and guidelines at America’s flagship universities. Educational Policy.

[67] Hosseini M, Gao P, Vivas-Valencia C (2025). A social-environmental impact perspective of generative artificial intelligence. Environmental Science and Ecotechnology.

