# Supplementary material for: Generative Artificial Intelligence for Qualitative Methods in Health Research: Rapid Review
Source: J Med Internet Res. 2026 Aug 31;28:e98551. doi: 10.2196/98551 (PMC13536981; doi:10.2196/98551)
Supplement: Supplementary file 2 [file jmir-v28-e98551-s001.docx]

Supplementary Appendix I

| **Appendix I.** Boolean logic syntax using all keyword combinations | | |
| --- | --- | --- |
| *Category* | *Keywords* | *Search Hedges* |
| Generative AI | ChatGPT  GPT-5  GPT-4  GPT-3.5  large language model  large language models  LLM  LLMs  generative AI  foundation model  foundation models  Claude  Gemini  LLaMA  Microsoft Co-Pilot  DeepSeek | "ChatGPT" OR "GPT-5" OR "GPT-4" OR "GPT-3.5" OR "large language model" OR "large language models" OR LLM OR LLMs OR "generative AI" OR  "foundation model" OR "foundation models" OR "Claude" OR "Gemini" OR "LLaMA" OR "Microsoft Co-Pilot" OR "DeepSeek" |
| Type of research | qualitative research  qualitative  thematic analysis  content analysis  grounded theory  interview*  focus group*  ethnograph*  qualitative data analysis | "qualitative research" OR qualitative OR "thematic analysis" OR "content analysis" OR "grounded theory" OR interview* OR "focus group*" OR ethnograph* OR "qualitative data analysis” |
| Qualitative Components | coding  data analysis  summar*  memo*  interpret*  agreement  reliability  workflow  efficiency | (coding OR "data analysis" OR summar* OR memo* OR interpret* OR agreement OR reliability OR workflow OR efficiency) |
| Exclusion | review  scoping review  rapid review  systematic review  literature review  narrative review | "review" OR "scoping review" OR "rapid review" OR "systematic review" OR "literature review" OR "narrative review" |
| **Note:** keywords with a “*” indicate a wildcard, which includes all any word/phrase allowing to find any variation of the keyword in the search engine. | | |
