# Supplementary material for: Generative Artificial Intelligence for Qualitative Methods in Health Research: Rapid Review
Source: J Med Internet Res. 2026 Aug 31;28:e98551. doi: 10.2196/98551 (PMC13536981; doi:10.2196/98551)
Supplement: Supplementary file 3 [file jmir-v28-e98551-s002.docx]

**Supplementary Appendix II**

**Appendix II**. Full Search Strategy

| **DATABASE – Web of Science** | **N - Searched 11-12-2025** |
| --- | --- |
| ***NUMBER OF ARTICLES IN DATABASE*** | **35** |
| **FINAL SINTAX**  TI=(("ChatGPT" OR "GPT-5" OR "GPT-4" OR "GPT-3.5" OR "large language model" OR "large language models" OR LLM OR LLMs OR "generative AI"  OR "foundation model" OR "foundation models" OR Claude OR Gemini OR LLaMA) AND ("qualitative research" OR qualitative OR "thematic analys*" OR  "content analys*" OR "grounded theory" OR interview* OR "focus group*" OR ethnograph* OR "qualitative data analys*") AND (coding OR "data analys*" OR summar* OR memo* OR interpret* OR agreement OR reliability OR workflow OR efficiency)) NOT TI=(review OR "scoping review" OR "rapid review" OR "systematic review" OR "literature review" OR "narrative review"). | SINTAX generated from  Web of Science |
| **BASE SINTAX**  ( "ChatGPT" OR "GPT-5" OR "GPT-4" OR "GPT-3.5" OR "large language model" OR "large language models" OR LLM OR LLMs OR "generative AI" OR  "foundation model" OR "foundation models" OR "Claude" OR "Gemini" OR "LLaMA" OR "Microsoft Co-Pilot" OR "DeepSeek" ) AND ("qualitative research" OR qualitative OR "thematic analysis" OR "content analysis" OR "grounded theory" OR interview* OR "focus group*" OR ethnograph* OR "qualitative data analysis”) AND (coding OR "data analysis" OR summar* OR memo* OR interpret* OR agreement OR reliability OR workflow OR efficiency) NOT ("review" OR "scoping review" OR "rapid review" OR "systematic review" OR "literature review" OR "narrative review") | 35 |
| ***SINTAX COMBINATIONS*** |  |
| **#1** - TI=(("ChatGPT" OR "GPT-5" OR "GPT-4" OR "GPT-3.5" OR "large language model" OR "large language models" OR LLM OR LLMs OR "generative AI" OR "foundation model" OR "foundation models" OR Claude OR Gemini OR LLaMA) | 226,564 |
| **#2** – TI=("qualitative research" OR qualitative OR "thematic analys*" OR "content analys*" OR "grounded theory" OR interview* OR "focus group*" OR ethnograph* OR "qualitative data analys*") | 1,772,289 |
| **#3** - TI=(coding OR "data analys*" OR summar* OR memo* OR interpret* OR agreement OR reliability OR workflow OR efficiency)) | 10,155,702 |
| **RESTRICTIONS** |  |
| NOT TI=(review OR "scoping review" OR "rapid review" OR "systematic review" OR "literature review" OR "narrative review"). | N/A |

| **DATABASE - PUBMED** | **N - Searched 11-12-2025** |
| --- | --- |
| ***NUMBER OF ARTICLES IN DATABASE*** | **372** |
| **FINAL SINTAX**  ("ChatGPT"[tiab] OR "GPT-5"[tiab] OR "GPT-4"[tiab] OR "GPT-3.5"[tiab] OR "large language model"[tiab] OR "large language models"[tiab] OR "LLM"[tiab] OR "LLMs"[tiab] OR "generative AI"[tiab] OR "foundation model"[tiab] OR "foundation models"[tiab] OR Claude[tiab] OR Gemini[tiab] OR LLaMA[tiab]) AND  ("Qualitative Research"[Mesh] OR qualitative[tiab] OR "thematic analys*"[tiab] OR "content analys*"[tiab] OR "grounded theory"[tiab] OR interview*[tiab] OR  "focus group*"[tiab] OR ethnograph*[tiab] OR "qualitative data analys*"[tiab)) AND (coding[tiab] OR "data analys*"[tiab] OR summar*[tiab] OR memo*[tiab]  OR interpret*[tiab] OR agreement[tiab] OR reliability[tiab] OR workflow[tiab] OR efficiency[tiab]) NOT (("review" OR "scoping review" OR "rapid review" OR  "systematic review" OR "literature review" OR "narrative review")) | SINTAX generated from  PubMed |
| **BASE SINTAX**  ( "ChatGPT" OR "GPT-5" OR "GPT-4" OR "GPT-3.5" OR "large language model" OR "large language models" OR LLM OR LLMs OR "generative AI" OR  "foundation model" OR "foundation models" OR "Claude" OR "Gemini" OR "LLaMA" OR "Microsoft Co-Pilot" OR "DeepSeek" ) AND ("qualitative research" OR qualitative OR "thematic analysis" OR "content analysis" OR "grounded theory" OR interview* OR "focus group*" OR ethnograph* OR "qualitative data analysis”) AND (coding OR "data analysis" OR summar* OR memo* OR interpret* OR agreement OR reliability OR workflow OR efficiency) NOT ("review" OR "scoping review" OR "rapid review" OR "systematic review" OR "literature review" OR "narrative review") | 372 |
| ***SINTAX COMBINATIONS*** |  |
| **#1** - ("ChatGPT"[tiab] OR "GPT-5"[tiab] OR "GPT-4"[tiab] OR "GPT-3.5"[tiab] OR "large language model"[tiab] OR "large language models"[tiab] OR  "LLM"[tiab] OR "LLMs"[tiab] OR "generative AI"[tiab] OR "foundation model"[tiab] OR "foundation models"[tiab] OR Claude[tiab] OR Gemini[tiab] OR  LLaMA[tiab]) | 25,236 |
| **#2** - ("Qualitative Research"[Mesh] OR qualitative[tiab] OR "thematic analys*"[tiab] OR "content analys*"[tiab] OR "grounded theory"[tiab] OR interview*[tiab]  OR "focus group*"[tiab] OR ethnograph*[tiab] OR "qualitative data analys*"[tiab)) | 859,668 |
| **#3** - (coding[tiab] OR "data analys*"[tiab] OR summar*[tiab] OR memo*[tiab] OR interpret*[tiab] OR agreement[tiab] OR reliability[tiab] OR workflow[tiab] OR efficiency[tiab]) | 3,500,222 |
| **RESTRICTIONS** |  |
| **#4 -** NOT ALL ( "review" OR "scoping review" OR "rapid review" OR "systematic review" OR "literature review" OR "narrative review" ) | N/A |

| **DATABASE – SCOPUS** | **N – Searched 11-12-2025** |
| --- | --- |
| ***NUMBER OF ARTICLES IN DATABASE*** | ***212*** |
| **FINAL SINTAX**  ( TITLE-ABS-KEY ( "ChatGPT" OR "GPT-5" OR "GPT-4" OR "GPT-3.5" OR "large language model" OR "large language models" OR LLM OR LLMs OR  "generative AI" OR "foundation model" OR "foundation models" OR "Claude" OR "Gemini" OR "LLaMA" OR "Microsoft Co-Pilot" OR "DeepSeek" ) AND TITLE-ABS-KEY ( "qualitative research" OR qualitative OR "thematic analysis" OR "content analysis" OR "grounded theory" OR interview* OR "focus group*" OR ethnograph* OR "qualitative data analysis" ) AND TITLE-ABS-KEY ( coding OR "data analysis" OR summar* OR memo* OR interpret* OR agreement OR reliability OR workflow OR efficiency ) AND NOT ALL ( "review" OR "scoping review" OR "rapid review" OR "systematic review" OR  "literature review" OR "narrative review" ) ) | SINTAX generated from  SCOPUS |
| **BASE SINTAX**  ( "ChatGPT" OR "GPT-5" OR "GPT-4" OR "GPT-3.5" OR "large language model" OR "large language models" OR LLM OR LLMs OR "generative AI" OR  "foundation model" OR "foundation models" OR "Claude" OR "Gemini" OR "LLaMA" OR "Microsoft Co-Pilot" OR "DeepSeek" ) AND ("qualitative research" OR qualitative OR "thematic analysis" OR "content analysis" OR "grounded theory" OR interview* OR "focus group*" OR ethnograph* OR "qualitative data analysis”) AND (coding OR "data analysis" OR summar* OR memo* OR interpret* OR agreement OR reliability OR workflow OR efficiency) NOT ("review" OR "scoping review" OR "rapid review" OR "systematic review" OR "literature review" OR "narrative review") | 212 |
| ***SINTAX COMBINATIONS*** |  |
| **#1** - TITLE-ABS-KEY ( "ChatGPT" OR "GPT-5" OR "GPT-4" OR "GPT-3.5" OR "large language model" OR "large language models" OR LLM OR LLMs  OR "generative AI" OR "foundation model" OR "foundation models" OR "Claude" OR "Gemini" OR "LLaMA" OR "Microsoft Co-Pilot" OR "DeepSeek" ) | 141,946 |
| **#2** - TITLE-ABS-KEY ( "qualitative research" OR qualitative OR "thematic analysis" OR "content analysis" OR "grounded theory" OR interview* OR "focus group*" OR ethnograph* OR "qualitative data analysis”) | 2,453,164 |
| **#3** - TITLE-ABS-KEY ( coding OR "data analysis" OR summar* OR memo* OR interpret* OR agreement OR reliability OR workflow OR efficiency ) | 13,146,606 |
| **RESTRICTIONS** |  |
| **#4 -** NOT ALL ( "review" OR "scoping review" OR "rapid review" OR "systematic review" OR "literature review" OR "narrative review" ) | N/A |
