# Supplementary material for: Generative Artificial Intelligence for Qualitative Methods in Health Research: Rapid Review
Source: J Med Internet Res. 2026 Aug 31;28:e98551. doi: 10.2196/98551 (PMC13536981; doi:10.2196/98551)
Supplement: Supplementary file 4 [file jmir-v28-e98551-s003.docx]

Supplementary Appendix III

| **Appendix III.** Data Extraction Variables | |
| --- | --- |
| Study ID (DOI) | As mentioned in the text. |
| Study Citation | As mentioned in the text. |
| Study Title | As mentioned in the text. |
| Year | As mentioned in the text. |
| Journal | As mentioned in the text. |
| Country | As mentioned in the text. |
| Institution Affiliation | As mentioned in the text. |
| Study Aim | As mentioned in the text. |
| Health Domain | Any health domain, such as chronic disease, health behaviors, and non‑communicable diseases, etc. |
| Study Setting | e.g., clinical, community, policy, academic, digital |
| Study Design | e.g., qualitative, mixed methods, experimental comparison |
| Qualitative Approach | e.g., thematic analysis, grounded theory, content analysis, etc. |
| Generative AI Used | e.g., ChatGPT, Llama, Gemini, etc. |
| Generative AI Task | e.g., coding, theme generation, summarization, classification |
| Reliability Assessment | How was reliability between human and AI outputs assessed. |
| Evaluation Metrics | Evaluation metrics were used to evaluate AI performance compared to other AI models or comparison with human analysis. |
| Ethics pertinent to the use of Generative AI | This could be related to IRB procedures and other ethical concerns (environmental, hallucinations of models) |
| Main Findings | Key findings of each study |
