# Supplementary material for: Generative Artificial Intelligence for Qualitative Methods in Health Research: Rapid Review
Source: J Med Internet Res. 2026 Aug 31;28:e98551. doi: 10.2196/98551 (PMC13536981; doi:10.2196/98551)
Supplement: Supplementary file 5 [file jmir-v28-e98551-s004.docx]

Supplementary Appendix IV

| **Appendix IV**. Main Findings of Generative AI Application, Reliability, Evaluation Metrics, and Ethics | | | | | | | |
| --- | --- | --- | --- | --- | --- | --- | --- |
| **Study** | **Study Aim** | **Qualitative Approach** | **Generative AI Tool** | **Specific Use of AI Tool** | **Reliability (Human vs. AI) & Evaluation Metrics** | **Main Findings** | **Ethics Pertinent to Application of Generative AI** |
| [1] | To investigate and compare the potential of two large language models to perform text coding in thematic analysis with the goal of reducing the time and effort required by human researchers. | Inductive Thematic analysis | ChatGPT-4; Open AI o1-preview | Text coding phase of thematic analysis, identifying and labeling segments of text with codes that summarize core content. | **Reliability:** Human coders independently examined each AI-generated code, compared it with the original text segment, and judged whether the code accurately captured the segment’s meaning or required revision; this review was then discussed jointly to reach consensus on accuracy.  **Evaluation Metrics:** Coding Density, Number of text-segments coded, Coding Consistency, Coding Accuracy. | Both models exhibit reduced coding comprehensiveness as document length increases and demonstrate low coding accuracy, primarily due to limitations in textual comprehension and reasoning. These findings highlight significant challenges in using LLMs for thematic analysis, underscoring the need for human oversight and rigorous validation to ensure analytic accuracy and validity. | The authors used de‑identified, IRB‑exempt student comments and protected confidentiality, stressing that human oversight is essential to prevent AI‑driven misinterpretation, bias, or inaccurate portrayals of qualitative data. |
| [2] | Explore ChatGPT’s performance when tasked with analyzing a qualitative transcript, including via a comparison of themes derived by ChatGPT and by experienced human researcher, as well as by an examination of ChatGPT’s engagement with other steps of the analytic process such as embedding derived themes into broader theoretical discourses. | Reflexive Thematic analysis (Performed by Human)    Grounded Theory (Charmaz) and Pope’s Five‑Step Framework (framework analysis) used to guide thematic analysis for ChatGPT | ChatGPT-3.5 (free version, mentioned explicitly by authors) | 1) Thematic coding of the transcript; 2) Generation of a qualitative codebook; 3) Extraction of illustrative verbatim style quotes for each theme; 4) Embedding of identified themes into broader theoretical lenses (socio-ecological model, Marxist lens, 5C vaccination model). | **Reliability:** Not explained in the methods.    **Evaluation Metrics:** No explicit evaluation metrics mentioned in methods, observations are made in results and discussion. | ChatGPT produced themes like those of an experienced researcher and added some nuanced categories. It generated a workable codebook and example quotes, though some required correction or were fabricated. Its theoretical interpretations were sometimes plausible but occasionally forced, such as mismatched model applications. Overall, ChatGPT can speed qualitative analysis and support triangulation, but it still requires human supervision, ethical oversight, and critical review of all outputs. | The authors note that no ethics approval was needed because they used a transcript they created and owned. They caution, however, that using ChatGPT with real qualitative data introduces privacy and data‑protection risks requiring IRB review and clear institutional policies. They advise avoiding uploads of sensitive data and conducting ethical and legal checks on consent and confidentiality beforehand. |
| [3] | To determine whether the information ChatGPT provides about HIV prevention and PrEP differs based on the user’s stated race, and to explore how the platform could inform public‑health education for Black women self‑educating about sexual health. | Inductive Content Analysis | ChatGPT-3.5 (free version, mentioned explicitly by authors) | To simulate user ChatGPT interactions on eight prompts about HIV prevention and PrEP, producing full conversation transcripts that were then compared for differences in Answers, Attitude, and Identity (A2I); content that was similar in content; and content that differed in content. | **Reliability:** Two analysts independently coded the transcripts and reconciled differences through discussion, acknowledging the inherent subjectivity of qualitative interpretation.    **Evaluation Metrics:** Differences in Answers, Attitude, and Identity; content that was similar in content; and content that differed in content. | ChatGPT consistently provided accurate, CDC‑aligned information on HIV prevention and PrEP. When the user was described as Black, responses included more detailed, culturally attuned guidance and specific financial‑assistance resources, whereas generic prompts (no race specified) produced broader, less targeted overviews. These variations show that ChatGPT’s outputs can shift based on perceived user identity, creating opportunities for more equitable, tailored messaging but also risks of unintended bias without careful monitoring. | The project was deemed non-human-subjects research, IRB approval was not required. Authors highlighted concerns about algorithmic bias, transparency, and privacy of user data when deploying ChatGPT for health information. |
| [4] | To demonstrate how artificial intelligence (ChatGPT) can be used to support the qualitative analysis of public comments on a newspaper article and to reflect on the researcher’s evolving experience from a naïve to an informed user of AI. | Autoethnographic case study employing a pragmatic, qualitative approach; the researcher first conducts a manual iterative thematic inquiry and then compares it with AI generated analysis. | ChatGPT-3.5 (free version, mentioned explicitly by authors) | The researcher uploaded all 44 comment texts into ChatGPT and asked the model to “analyse the text of the comments for themes.” ChatGPT produced initial codes, grouped them into broader themes, and delivered a final set of five themes. | **Reliability:** assessed by conducting a full manual ITI analysis first, then running the same data through ChatGPT, and finally comparing the two sets of themes. The researcher examined overlap, noted where AI missed the positive/negative split, and discussed reasons for discrepancies.    **Evaluation Metrics:** No format evaluation metrics were reported; evaluation relied on qualitative comparison of theme content. | ChatGPT generated five themes that largely overlapped with the six manually derived themes but did not separate positive and negative sentiment. The differences were attributed to the inclusion of satirical/irrelevant posts and the lack of a targeted research question in the AI prompt. The researcher concluded that AI could support qualitative analysis when used after a thorough manual review, that specific prompts improve AI output, and that ethical transparency and researcher expertise are essential to maintain trustworthiness. | The researcher emphasized transparent reporting of AI use and taking responsibility for the analysis. Manual coding was completed first to avoid biasing AI outputs, and AI was used only with a clear research question and precise instructions. Because the data were publicly posted comments, ethical risk was low, but the researcher still addressed honesty, data manipulation, and the ethics of re‑analyzing data with AI. |
| [5] | To demonstrate how LLMs can augment or replace human coders in content‑analysis projects, replicate Feldman et al.’s (2015) climate‑change newspaper study, validate LLM‑generated codes against human coding, and show how the approach scales to a ten‑fold larger corpus and to a German‑language dataset. | Content Analysis | Llama 3.1 (6‑bit quantized) | Automated classification (coding) of each newspaper article for climate change impacts, frames, timing, location, action types, and efficacy; the LLM acted as Instructor, Classifier, and Judge within an agentic workflow. | **Reliability:** ICR was assessed with Krippendorff’s α; LLM‑human agreement reached α ≥ 0.70, for most constructs after iterative prompt refinement.  **Evaluation Metrics:** Krippendorff’s α – the main indicator of intercoder reliability. The prompt refinement process was guided toward achieving α ≥ 0.70. Reliability was calculated on the full sample (training + validation) and separately on the held-out validation set to assess overfitting. Sensitivity (positive identification rate) – the proportion of coded variables that the LLM flagged as present. | A locally run Llama 3.1 replicated human coding with similar reliability, enabling cost‑effective, multilingual, large‑scale text analysis. When the LLM-pipeline expanded from 642 to 3,274 articles, reliability remained stable, and coding became richer: for example, distinguishing impact from action variables. The model was slightly more sensitive than human coders, yielding higher positive coding rates without altering conclusions. However, reliability for complex constructs like “efficacy” stayed lower (α ≈ 0.62), highlighting ongoing limitations and the need for human oversight. Overall, the agentic workflow, using meta‑prompting, chain‑of‑thought, and iterative refinement, improved consistency, and interpretability. | Local deployment ensured data privacy and compliance with copyright; human-in-the-loop oversight mitigated hallucinations and bias; the authors discuss broader ethical issues such as transparency, accountability, and the environmental impact of model computation. |
| [6] | To evaluate the consistency of themes, the reliability of coding, and the time required for inductive and deductive thematic analyses when performed by generative AI (ChatGPT‑3.5 and Google Bard) compared with human coders. | Inductive and deductive thematic analysis | ChatGPT-3.5, Google Bard (Both free versions, mentioned explicitly by authors) | The AI models were prompted to (1) generate a codebook, (2) apply the codebook to label each SMS message data, and (3) extract themes for both inductive and deductive analyses of the 40 messages. | **Reliability:** ICR was calculated as percent agreement between human coders and each AI system for shared themes for deductive and inductive coding.    **Evaluation Metrics**: Theme consistency, defined as the percentage of human-identified themes reproduced by AI; intercoder reliability, measured as percent agreement; and time efficiency, assessed by the minutes required for analysis. | The themes generated by GenAI (both ChatGPT and Bard) were consistent with 71% (5/7) of the themes identified by human analysts following inductive thematic analysis. The consistency in themes was lower between humans and GenAI following a deductive thematic analysis procedure (ChatGPT: 6/12, 50%; Bard: 7/12, 58%). The overall ICR (percent agreement in coding) of themes shared between human coders and GenAI models (inductive: 31/66, 47% and 20/54, 37%; deductive: 22/59, 37% and 21/58, 36%, respectively) was fair to moderate. In addition, GenAI models were significantly less resource-intensive, as they took an average of 97% less time (20 vs 567 min) for qualitative analysis compared to human coders. ChatGPT and Bard performed similarly to each other across both types of thematic analysis. | The authors highlighted privacy concerns (use of participant—generated text with third-party AI services), potential bias, “hallucinations,” informed consent challenges for at-risk populations, and the need for ethical oversight when employing GenAI in human-participant research. Authors mention that GenAI can accelerate qualitative work, but lower coding reliability suggests that hybrid human-AI workflows are needed for nuanced interpretation. |
| [7] | Evaluate the feasibility and performance of ChatGPT for inductive coding of semi‑structured interviews with maternity‑care providers and propose a computational workflow that automates coding while comparing AI‑generated codes with manually created ones. | Thematic Analysis (inductive) guided by Braun & Clarke’s Framework | ChatGPT 4 Model (accessed via web interface and API) | Automated inductive coding of interview transcripts: (1) dialogue filtering to locate relevant question answer pairs, (2) code generation (extracting and summarizing key topics), (3) aggregation of codes into categories and themes. | **Evaluation and Evaluation Metrics:**  1) Human-centered approach: Two domain experts independently reviewed AI-generated codes, marking each as “accepted” when it matched or aligned with human codes; overall agreement yielded an accuracy rate.    2) Machine-based approach: Semantic similarity between AI and human codes was quantified using BERT (Bidirectional Encoder Representations from Transformers) embeddings and cosine similarity. | The model demonstrated strong overall performance, achieving an accuracy rate of 85.15% across all questions, with individual question accuracy ranging from 66.67% to 100%. More than half of the questions (11 in total) achieved coding accuracy rates of 90% or higher, including eight questions with perfect (100%) accuracy, while only three questions fell below 80%. Review of these lower-performing items indicated that highly descriptive responses led ChatGPT to extract more generalized information. Despite this, the inductive coding results were considered reliable, highlighting the model’s promise in supporting qualitative coding. In terms of efficiency, coding time decreased substantially from approximately 30.5 hours for human analysis to about 6 total hours (≈5 hours setup and ≈1 hour AI processing), representing an 81% reduction. Stability analyses showed highly consistent outputs across reruns (average semantic similarity >0.95), and semantic alignment between AI- and human-generated codes was high (overall similarity 0.795; best case 0.879). Nonetheless, limitations included occasional over-generalization of detailed responses, limited domain-specific terminology, sporadic hallucinations, and the continued need for human expert review. | Recommendations for data de-identification, secure access controls, and future privacy, preserving techniques (differential privacy, secure multiparty computation, machine unlearning). |
| [8] | To evaluate ChatGPT as an augmentative tool for qualitative interview analysis by replicating a 2019 causal-loop diagram (CLD) of obesity-prevention interventions and comparing AI-generated results with the original human-derived model. | System-dynamics based CLD construction derived from coded interview variables, links, and feedback loops. | ChatGPT-4 | Prompted to extract key variables from interview texts Identify causal links between variables. Propose and enumerate feedback loops, producing a full CLD. | **Reliability:** The authors compared the AI-generated loops (31 total) with the original CLD, focusing on overlap around “Motivation of stakeholders” and noting where AI added new employee—level loops.  **Evaluation Metrics**: Degree of overlap with original loops (e.g., replication of R1). Presence of novel loops (e.g., employee-time-constraints loop). Qualitative assessment of directional accuracy and contextual fidelity. | ChatGPT efficiently extracted variables and generated a richer set of feedback loops, uncovering nuanced employee-level dynamics absent from the original model. The AI produced CLD matched key managerial loops but exhibited occasional directional errors and limited contextual nuance. Authors conclude AI should serve as a complementary, augmentative aid rather than a replacement for human qualitative analysis, emphasizing rigorous cross-validation and transparency. | Data ownership and privacy concerns under the GDPR when uploading interview transcripts to a proprietary platform. Potential bias from training data and the “black box” nature of the model. Need for version reporting and cross checking to ensure reproducibility. |
| [9] | To evaluate the feasibility, strengths, and limitations of ChatGPT-4 in analyzing qualitative Japanese interview data by directly comparing its performance with that of experienced human researchers. | Thematic Analysis, Grounded Theory, Framework Analysis | ChatGPT-4 | The model was tasked with conducting full thematic analyses of each transcript, generating codes and themes autonomously. Two established approaches, Grounded Theory and Framework Analysis, were used to test ChatGPT’s flexibility and consistency, assessing how well it adapted to different thematic analysis methodologies. | **Reliability**: assessed by comparing thematic agreement rates between ChatGPT and human researchers; agreement exceeded >80 % for descriptive themes and fell to ≈30 % for culturally nuanced themes.    **Evaluation Metrics**: Descriptive agreement rates (percentage of themes identified by both AI and humans) and frequency counts of theme occurrence were reported. | ChatGPT 4 reliably identified frequently occurring, descriptive themes (e.g., “personal experience of a sacred moment,” “building relationships”) with >80 % agreement to human coders. Its performance declined markedly for themes requiring deep cultural or emotional interpretation (e.g., “difficult to answer, no experience of sacred moments,” “fate”), achieving only ≈30 % agreement. The authors conclude that AI can serve as an efficient auxiliary tool for extracting surface level themes in Japanese language qualitative data but cannot replace human expertise for nuanced, culturally embedded interpretations. | IRB approval and informed consent were obtained. All transcripts were fully anonymized and stored securely. The study highlighted the necessity of human oversight to mitigate AI related biases and ensure ethical use of generative models in qualitative research. |
| [10] | The study aimed to investigate if an LLM achieves sufficient agreement with a researcher in the deductive coding of interview data obtained in a psychosocial autopsy and can summarize data of adequate quality to be feasibly integrated with qualitative research procedures. | Deductive coding using a predefined codebook; summary quality assessed with the Constant Comparative Method. | LLaMA-3 70B instruct version | Binary classification of whether a predefined code is present in short transcript fragments. Sliding‑window classification to locate code‑relevant segments across entire transcripts. Summarization of all identified segments per code for downstream qualitative analysis. | **Reliability**: Accuracy and intercoder agreement (Cohens Kappa) were calculated. Landis and Koch propose criteria for agreement between qualitative researchers, with a Cohens Kappa of less than 0 as indicating no agreement, between 0 and 0.20 as slight, 0.21 and 0.40 as fair, 0.41 and 0.60 as moderate, 0.61 and 0.80 as substantial, and 0.81 and 1 as nearly perfect agreement.  **Evaluation Metrics**: 1) Accuracy, sensitivity, specificity for each code (binary and sliding window tasks). 2) Cohen’s κ for intercoder reliability. 3) Relative code frequency ratios (LLM / researcher). 4) Qualitative rating of LLM generated summaries (good / adequate / poor). | The LLM attained 84 % accuracy (κ = 0.68) on binary coding, performing best on concrete codes (e.g., suicide method, location) and poorly on behavioral/interpersonal codes, generating many false positives. Sliding window coding accuracy dropped to 67 %; the model tended to over label low frequency codes (factor ≈ 1.6). Summaries were rated “adequate” or “good” in ≈ 80 % of cases; 1.3 % of summarized fragments contained hallucinated or mis coded content. The LLM processed all 38 interviews in ~8 days (≈ 5 h per interview), compared with ~4 months of manual coding. Researchers recommend a collaborative workflow: LLM performs initial deductive coding, followed by researcher review, inductive coding, and active learning feedback to mitigate bias and hallucination. | The study received ethics approval, with informed consent obtained and data access restricted for privacy. How AI use was evaluated by the committee is unclear. The authors note that their own created transcript required no additional approval but warn that applying ChatGPT to real qualitative data raises privacy and data‑protection concerns needing IRB review and clear institutional policies. They recommend avoiding uploads of sensitive data and conducting ethical and legal checks on consent and confidentiality beforehand. |
| [11] | The dual purposes of this study were (a) to compare the data analysis process and outcomes of novice qualitative researchers and ChatGPT-4 and (b) consider advantages and disadvantages of using ChatGPT-4 for qualitative coding. | Inductive Content Analysis | ChatGPT-4 (accessed through a paid subscription) | Automated line-by-line coding of the 60 open-ended responses, reduction of redundant codes, organization into parent/child categories, and generation of themes and narrative summaries. | **Reliability:** Human and AI outputs were compared by examining code counts, category and theme structures, and time required; the study highlighted differences in transparency and depth rather than formal statistical reliability measures.  **Evaluation Metrics**: Number of initial and final codes; Number of parent categories; Number of themes; Total coding time; Qualitative depth; contextual richness; and transparency (subjective expert assessment). | ChatGPT-4 completed the QCA dramatically faster than novice human coders while producing a comparable quantity of codes, categories, and themes; human coders generated richer, more contextually nuanced categories and themes and provided a transparent, auditable coding trail; AI outputs lacked depth, sometimes blurred the distinction between codes, categories, and themes, and offered limited transparency; the study concludes that AI can accelerate qualitative coding but should be paired with human expertise to preserve rigor, coherence, and ethical standards. | Need for human oversight to ensure methodological rigor, auditability, and interpretive nuance; concerns about opaque analytic pathways and lack of step-by-step justification; discussion of potential bias, environmental impact of AI, and responsibility of developers to improve transparency and explainability. |
| [12] | To understand how LLMs can help analyze expert textual data and to investigate the use of LLMs to scale the interpretive stage of thematic analysis in qualitative health care research. | Inductive Thematic Analysis | ChatGPT-3.5 | Prompted the LLM to generate themes and subthemes for each LDA identified topic, thereby performing thematic summarization, and labeling of the topics. | **Reliability:** A total of 2 authors independently classified the level of agreement on each topic against the themes and subthemes generated by ChatGPT, using the 7-point agreement scale. The authors then met to compare assessments and resolve disagreements. Detailed ratings of alignment, convergence, coherence, and complementarity were also determined.  **Evaluation Metrics**: Overall agreement percentage; Taxonomic agreement at theme level; Sub-theme agreement: Alignment, convergence, coherence, and complementarity scores. | Substantial agreement (80%) between LLM generated and human generated themes, indicating that LLMs can reliably automate thematic interpretation. LLM derived subthemes added depth and uncovered complementary insights, but occasional misclassifications occurred, especially for divergent sub themes. LLMs tended to over generalize or over fit certain topics, highlighting the need for researcher oversight and contextual framing. | The study used only publicly available, anonymized forum posts; no identifiable private information or direct interaction with individuals, so institutional review board approval was deemed unnecessary. |
| [13] | We aimed to compare themes generated by ChatGPT (3.5 and 4.0) with traditional human analysis from in-depth interviews. Our focus on the comparison with traditional human analysis represents a novel contribution to the evolving landscape of AI-driven research methodologies and sheds light on ChatGPT’s reliability and appropriateness for practical use with traditional human analysis. | Thematic Analysis | ChatGPT-3.5 and ChatGPT-4 | Performing thematic analysis of interview transcripts about patient experience at a community eye clinic. Summarizing each transcript and generating themes and subthemes from the text, in batches of transcript pages. | **Reliability:** Regrouping ChatGPT-generated subthemes under the predefined human-generated theme definitions. Calculating concordance as “the number of correct themes generated by ChatGPT divided by the number of themes generated by the researcher.” Comparing numbers and types of irrelevant (non-related) subthemes produced by ChatGPT.  **Evaluation Metrics**: Concordance of themes between ChatGPT and the human researcher, expressed as counts and percentages. Number of irrelevant/non-related subthemes across the three interviews. Time taken to generate themes per transcript. Additional time required for regrouping ChatGPT subthemes into the researcher’s themes. | ChatGPT 3.5 and 4.0 produced themes with moderate to good agreement with a human researcher (average concordance 83.7%, range 66.7–100%). Both versions of ChatGPT were ~20 times faster than manual analysis for initial theme generation (about 11.5–11.9 minutes vs 240 minutes per transcript), and even after adding regrouping time, using ChatGPT remained substantially faster (51.5 minutes for 3.5 and 41.9 minutes for 4.0 vs 240 minutes). ChatGPT 4.0 generated fewer irrelevant subthemes than 3.5 (13 vs 20), suggesting somewhat better contextual relevance, though 4.0 took slightly longer to run. ChatGPT can support rapid preliminary analysis but cannot replace human researchers, who must interpret nuances and ensure contextual fit. It works best as a complementary tool. | The study IRB approval and written consent. Using cloud-based AI poses risks to data protection because inputs may be stored or used for training, requiring strict handling of patient information. Human oversight is essential to ensure accuracy, context, and ethical use. AI bias is possible because many models are trained on predominantly White, higher‑SES data, which may misalign with Asian or under‑represented groups. AI-assisted analysis may also be unsuitable when deep emotional or nuanced human interpretation is needed. |
| [14] | Study aimed to test if Microsoft’s Copilot could perform a content analysis on interview data using Graneheim and Lundman’s method comparable to human analysis. | Content Analysis (Manifest & Latent) | Microsoft Copilot | Identifying and extracting meaning units from each interview transcript, condensing the meaning units into shorter, essential statements, assigning codes to the condensed meaning units, developing sub-themes from the coded data, and conducting a cross-case analysis to create overarching themes across all six interviews. | **Reliability:** The manual analysis served as the gold‑standard; Copilot outputs were systematically compared using a three‑color coding scheme (green = exact match, yellow = same meaning, red = no match) for meaning units, codes, sub‑themes, and themes.  **Evaluation Metrics**: Degree of consensus between Copilot and manual analyses (color‑coded matches), completeness of meaning units, accuracy of coding, similarity of sub‑themes and overarching themes, and length/detail of the final output. | Copilot reproduced the manifest content (meaning units, codes, sub-themes, and overarching themes) with high resemblance to the manual analysis. Latent (interpretive) content was less nuanced; Copilot generated shorter, less detailed outputs and occasionally over-interpreted when given study context or objectives. Performance was best when Copilot was prompted only with the methodological framework (Graneheim & Lundman) and not with additional context or objectives. The authors conclude that Copilot can serve as a second-pass, resource-saving tool for early, manifest-content coding, but human oversight remains essential for interpretive analysis. | Use of a company-protected Copilot version ensured that data were not stored for model training; informed consent was obtained from participants; GDPR-compliant data handling and anonymization were applied. |
| [15] | The study aimed to identify key themes that emerged from participants’ free-text responses to a large nationally representative vignette survey about surrogate decision-making in end-of-life care and determine if an advanced AI chatbot could assist in accurately and efficiently performing qualitative analyses. | Inductive Thematic Analysis | ChatGPT-4o | Prompted the model to apply the human generated codebook to code the entire sample, generate keyword frequencies, derive major themes and subthemes, and select illustrative “quotable” quotes. | **Reliability:** Assessed by comparing AI-generated keyword counts with counts from NVivo qualitative software, and having human analysts cross-validate AI-generated themes and quotes against the original transcripts.  **Evaluation Metrics**: Keyword count agreement between AI and NVivo. Theme overlap between human-derived and AI-derived thematic sets. Error typology for AI-generated quotes. Time efficiency comparing manual coding and AI theme generation. | ChatGPT-4o reproduced the human-derived themes with high fidelity and identified additional lower-prevalence themes (e.g., decision-making challenges, ethical considerations, patient dignity). AI keyword frequencies matched those from NVivo (e.g., “life” 2.51 % vs. 2.48 %). AI-generated themes were primarily descriptive, mirroring participants’ explicit statements rather than latent meanings. Human validation uncovered three error types (quote fabrication, paraphrasing, merging), highlighting the need for rigorous post-processing. Overall, AI dramatically reduced analytic time while maintaining comparable content validity, suggesting that generative AI can effectively augment qualitative analysis of large free-text datasets when paired with systematic human verification. | The study was exempt by the Johns Hopkins IRB; data were fully anonymized and no personal health information was provided to the AI. The authors discuss broader ethical concerns such as data-privacy risks when uploading text to commercial AI services and recommend institutional review and the use of enterprise-grade AI solutions. |
| [16] | To explore whether LLMs can assist in refining complex medical definitions by evaluating three advanced LLMs’ ability to interpret, critique, and build upon existing definitions of clinical obesity, especially the Lancet Commission’s definition. | Authors mention all model outputs were reviewed qualitatively by a single expert in obesity medicine for accuracy, originality, and alignment with authoritative guidelines. | **GPT-4o** via OpenAI’s ChatGPT Plus environment, **Gemini 1.5 Pro** through Google’s experimental API, and **Grok 3** through its open beta web interface. | Each model prompted with five structured questions about the definition, diagnostic criteria, pitfalls, and recommendations for clinical obesity (baseline round), re-prompted with the full 40-page Lancet Commission report uploaded as contextual reference (contextual round), and asked to generate an improved, comprehensive definition of clinical obesity (generative round). | **Reliability:** Model outputs were reviewed by a senior medical expert for accuracy, originality, and concordance with authoritative guidelines; no quantitative inter-rater reliability testing was performed.  **Evaluation Metrics**: Factual accuracy, adherence to current scientific standards, responsiveness to injected context, originality, and internal coherence of the generated definitions. | Without contextual guidance, LLMs produced generalized definitions largely reflecting public domain sources. Supplying the Lancet Commission report yielded more precise, conceptually consistent, and clinically relevant outputs. In the generative phase, all models proposed refined definitions that addressed recognized gaps in existing frameworks, emphasizing functional impairment, psychosocial dimensions, and the limitations of body mass index as a diagnostic criterion. When anchored to authoritative, domain-specific evidence, large language models can serve as adjunctive tools for knowledge synthesis, clarifying and strengthening complex medical constructs. Integrating human expertise with artificial intelligence under transparent and ethically governed conditions may enable the development of more inclusive, adaptive, and evidence-based clinical definitions. | Authors emphasized that responsibility for interpretation remains with human experts; highlighted risks of hallucinations, bias, and stigmatizing language; called for transparent reporting, expert oversight, and ethical safeguards when integrating AI into medical definitions. |
| [17] | The aim of the study was to understand how to enhance support mechanisms that promote longer treatment stays with the view to improving well-being outcomes. | Thematic Narrative Analysis | ChatGPT-4o | Prompt-driven thematic extraction from interview transcripts. Generation of first-person narrative vignettes based on identified themes. Assisted coding and organization of qualitative data (theme identification, vignette drafting). | **Reliability:** Researchers triangulated AI-generated themes with manually coded themes, cross—checking each transcript; discrepancies were resolved through discussion and revision. Member-checking with participants ensured the AI-produced vignettes faithfully reflected their voices.  **Evaluation Metrics:** Convergence/divergence of theme sets (human-coded vs. AI-generated). Qualitative assessment of vignette accuracy via participant member-checking. | AI-assisted narrative analysis substantially accelerated the synthesis of long interview transcripts into concise, participant-authentic vignettes. AI-generated themes uncovered patterns that sometimes differed from manual coding, prompting richer interpretation after triangulation. Combining human insight with ChatGPT output enhanced methodological rigor while preserving the depth and emotional nuance of participants’ stories. The approach demonstrated that generative AI could serve as a complementary tool in qualitative health research, improving efficiency without sacrificing ethical standards or data fidelity. | Ethics approval obtained and explicit consent for AI use was secured. Transcripts were de-identified; ChatGPT’s memory was disabled to prevent data retention. Member-checking provided participants control over their narratives and mitigated privacy risks. Transparency about AI involvement was maintained throughout the research process. |
| [18] | To evaluate the effectiveness of off-the-shelf and RAG-enhanced LLMs in delivering guideline-adherent nutrition information for CVD prevention, we assessed 3 off-the-shelf models (ChatGPT-4o, Perplexity, and Llama 3-70B) and a Llama 3-70B+RAG model. | Expert reviewers qualitatively assessed model outputs for appropriateness, reliability, harm, readability, and guideline adherence. | ChatGPT-4o, Perplexity, Llama 3-70B, and Llama 3-70B+RAG. | Generating answers to 30 nutrition questions (developed and vetted by a preventive cardiology dietitian) to assess guideline adherence and informational quality. | **Reliability:** Repeating each question three times per model and comparing consistency of responses. Calculating inter‑rater agreement among three expert reviewers using Cohen’s κ (Cohen coefficient).  **Evaluation Metrics:** Model responses were evaluated by 3 expert reviewers against benchmark CV guidelines for appropriateness, reliability, readability, harm, and guideline adherence. Mean scores were compared using ANOVA, with statistical significance set at P<.05. Interrater agreement was measured using the Cohen k coefficient, and readability was estimated using the Flesch-Kincaid readability score. | The RAG-enhanced Llama 3 model achieved significantly higher scores for reliability, appropriateness, and guideline adherence compared with off-the-shelf models. No harmful content was detected in the RAG-enhanced model’s outputs, whereas off-the-shelf models produced several potentially unsafe recommendations. Responses were less readable for the RAG model versus the off-the-shelf models, reflecting more technical language drawn directly from guidelines. | The study was classified as non‑human‑participant research and received an exempt determination from the Stanford IRB. Ethical focus included monitoring for harmful or misleading nutrition advice, ensuring citation accuracy, and addressing privacy/HIPAA concerns when deploying LLMs in clinical contexts. |
| [19] | LLMs face challenges in inductive thematic analysis, a task requiring deep interpretive, domain-specific expertise. We evaluated the feasibility of using LLMs to replicate expert-driven thematic analysis of social media data. | Inductive Thematic Analysis | GPT-3.5-turbo, GPT-4o (used through API), DeepSeekV3 (deepseek-v3-0324-ud-q2_k_xl), Gemma3 (gemma-3-27b-it), and Llama3 (llama3-70b). | LLMs were prompted (zero‑, one‑, and two‑shot prompting) to classify each Reddit post as “relevant” (1) or “not relevant” (0) for each of the 12 themes, effectively performing automated thematic coding. | **Reliability:** Assessed by comparing model-generated theme assignments to the expert-coded gold standard on the validation set, reporting agreement metrics (accuracy, precision, recall, F1) and confidence intervals.  **Evaluation Metrics:** Accuracy, Precision, Recall, F1 score. | ChatGPT-4o with 2-shot prompting achieved the highest performance, outperforming other models. DeepSeek-V3 performed comparably but slightly lower. Model-derived theme proportions closely matched expert distributions for the most prevalent themes. Few-shot prompting approaches can automate inductive thematic analysis at scale, reducing expert labor while maintaining reasonable agreement. Limitations include moderate precision and recall, dependence on predefined themes, and variability across zero-shot versus few-shot prompting. While LLMs can identify themes with reliable performance, inductive thematic analysis still requires human initiation and specification of themes to facilitate LLM-driven inference. | IRB approval obtained. All Reddit data were publicly available, data sharing subject to a use agreement. Discussion of potential bias amplification, need for human oversight, and cost considerations for API-based models. |
| [20] | The primary objective of this study was to establish a baseline by exploring and reflecting on how base LLMs, without reasoning or deep research capabilities, may support researchers in the analytical process of RTA by reanalyzing an interview text from the health science field. The secondary objectives were to reflect on and recommend a prompt strategy that could enhance LLMs’ analytical support, identify potential challenges and weaknesses, and foster engagement and reflections among qualitative researchers regarding these threats and possibilities. | Reflexive Thematic Analysis | Offline Mistral‑7B (7 billion‑parameter model) | Summarization for the familiarization phase (150- and 300-word summaries). Generation of semantic codes (keyword-style coding). Attempted generation of initial themes, sub-themes, and refined themes. Linking the interview text to Donabedian’s quality-of-care theory and to patient-safety-culture frameworks. Drafting of write-up sections and clinical-implication suggestions. | **Realibility:** The authors compared LLM outputs to the original human-conducted RTA, using iterative reflexive memos, error-checking, and direct content—matching rather than formal statistical reliability metrics. Discrepancies (e.g., missed essential topics, fabricated quotes) were documented and corrected by the researchers.  **Evaluation Metrics:** Content coverage (whether essential topics were captured). Accuracy of generated quotes and citations (identifying fabricated quotations). Alignment with theoretical frameworks (correct vs. irrelevant theory linkage). No numerical reliability indices were reported. | The LLM produced only surface-level summaries and failed to generate interpretive, abstracted themes; many codes were mere keywords. Misinterpretations occurred, including missing irony, fabricating participant quotes, and linking to irrelevant theories. Translation sensitivity was evident: English-language prompts yielded better but still limited responses compared with Norwegian inputs. LLM support did not reduce analysis time; additional verification steps increased workload. A tightly controlled prompt strategy and thorough human verification were essential; high-quality RTA remains fundamentally a human-driven, reflexive activity. | Ethical approval from the Norwegian Agency for Shared Services in Education and Research and compliance with the Helsinki Declaration. Strict offline processing to protect participant privacy and GDPR compliance. Discussion of risks such as hallucinations, fabricated data, and potential clinical harm; emphasis on human oversight and audit trails. |
| [21] | The objective of this study was to explore the medication management-related needs of young adults with asthma via a pilot feasibility study. Study aimed to understand how to best assist young adults with asthma self-management and to identify potential areas where digital health interventions can provide support. Further aimed to understand the comparative outcome of human versus multiple AI platforms in performing thematic analysis. | Inductive Thematic Analysis | Gemini, Microsoft Copilot, and ChatGPT | The AI platforms were prompted to read the de‑identified transcripts, generate codes, categorize them into emergent themes, and provide sample quotes; essentially performing automated coding and theme generation. | **Reliability:** assessed by comparing the overlap of underlying concepts between human-derived themes and AI-derived themes, examining label similarity, counting the number of themes, and noting divergent interpretations (e.g., “embarrassment”).  **Evaluation Metrics:** Degree of conceptual overlap between human and AI themes, number and labeling of themes produced, time required for analysis, and identification of divergent or over-interpreted labels. | Human (performed using NVivo) and AI analyses yielded highly overlapping concepts; despite different labels, the same four support domains (clinician support, social support, digital self-management, educational support) emerged. AI completed thematic analysis within minutes, dramatically faster than the multi-month human process. AI occasionally over-interpreted nuances and identified themes not present in the human analysis. The study demonstrates that AI can efficiently assist thematic analysis in qualitative health research, provided that expert oversight addresses privacy, interpretive limits, and potential bias. | Only fully anonymized, non-sensitive transcript excerpts were input into AI tools. No personally identifying information was shared with the models. Data were stored on secure, access-controlled servers in compliance with IRB and data-privacy standards. |
| [22] | Study aimed to: (1) To test the LLM’s ability to provide meaningful analysis; (2) To quantitatively assess the LLM’s performance compared to manual coding; (3) To qualitatively evaluate the LLM’s potential to augment the manual coding process and understand the challenges and limitations in the process. | Immersion/Crystallization framework for manual coding; LLMs were used for descriptive contextual coding of pre-defined categories. | Claude 3.5 Sonnet and ChatGPT (OpenAI-API) | Automated categorical coding of eight contextual variables (age, gender, ethnicity, receiver identity, surprise, illness, setting, specialty) in 166 Hebrew narratives; also assisted in Python script development and manuscript wording. | **Reliability:** Agreement between LLM and human coders was quantified using Cohen’s κ, overall accuracy, and F1‑score for each category.    **Evaluation Metrics:** Cohen's K, overall accuracy, and F1 score, percentage of differing codes per category was also reported. | LLMs accurately reproduced human coding for straightforward contextual data such as age, gender, ethnicity, illness, and physician specialty with high agreement and accuracy. Substantial disagreements occurred for nuanced categories such as receiver’s identity, surprise, and setting, reflecting difficulty in interpreting implicit or ambiguous information. Overall, LLMs are reliable for extracting explicit descriptive information but fall short on interpretive, emotionally laden, or context-dependent coding. The authors recommend a hybrid “LLM-in-the-loop” workflow in which the LLM performs initial descriptive coding, followed by human expert review and thematic deepening to preserve interpretive depth. | Approved by the Tel Aviv University Ethics Committee, with no additional consent needed for secondary analysis. Narratives were de-identified before AI processing. Claude 3.5 was selected for its data-handling policies, including no use of customer data for training, limited data retention, and compliance with SOC 2 and HIPAA standards. |
| [23] | To introduce and illustrate a three-step “query-based analysis” workflow that uses ChatGPT to generate themes, sub-themes, and illustrative quotations from qualitative health data, thereby streamlining parts of traditional thematic analysis. | Query-Based Analysis | ChatGPT-3.5 (API) together with ChatDOC pre-processor. | Broad, undirected queries to obtain a list of core themes; Follow-up queries to elaborate sub-themes; Retrieval of illustrative quotations linked to each theme. | **Reliability**: The researcher manually inspected AI‑generated themes and quotations, checking for “hallucinations,” bias, and alignment with the original data . No formal inter‑rater statistics were reported; the process relied on expert familiarity with the transcripts.  **Evaluation Metrics:** Time efficiency: Mastering the QBA workflow required less than two hours versus several days for traditional CAQDAS coding. Thematic concordance: AI-derived themes were compared with those obtained via manual coding; overlap and coherence were judged by the researcher. Validity checks: Human judgment was used to confirm that AI-produced themes captured the research goals, and the author suggests future head-to-head comparisons with manual coding as a quantitative validation step. | QBA successfully generated five overarching themes and ten overlapping sub-themes from the caregiver focus-group data. The AI-driven workflow streamlined steps 3–5 of Braun and Clarke’s six-step thematic analysis, reducing analyst time dramatically while preserving interpretive depth. The author concludes that QBA offers a viable, efficient alternative to traditional coding, but emphasizes the need for comparative validation studies to confirm equivalence with manual methods. | Full de‑identification of transcripts before AI processing; the data‑retention option in ChatGPT was disabled to prevent the dataset entering the model’s training set. Discussion of AI‑related risks (hallucinations, bias) and the necessity of a human reflexive loop to mitigate them. |
| [24] | The objective of this study was to examine the content provided by AI platforms regarding the availability of accurate, actionable information and the complexity of responses regarding HIV-related health information. | Inductive content analysis | ChatGPT 3.5 (free), ChatGPT 4.0 (paid at time of study), Google Bard [now Gemini], HIV.gov Chatbot | The AI platforms were asked 38 question prompts concerning HIV medication safety, efficacy, and side-effects; responses were recorded verbatim for analysis. | **Reliability**: Two independent human raters coded all transcripts, and a third coder resolved discrepancies, providing inter-rater reliability for the qualitative coding. Consistency of AI output over the four weeks was also assessed, including variation in code counts.    **Evaluation Metrics:** Total number of coded themes per response used as a measure of comprehensiveness. Total number of codes generated by each platform. Week-to-week consistency of code counts. Frequency of citation inclusion. | All platforms affirmed the safety and efficacy of HIV antiretroviral medications and consistently recommended consulting a healthcare professional. Google Bard delivered the most comprehensive content with the greatest variability across weeks. ChatGPT 4.0 provided the most consistent and relatively comprehensive responses. The HIV.gov Chatbot gave the shortest responses but included the highest proportion of citations and hyperlinks. Side effects were most frequently described as organ or bone and gastrointestinal issues; Bard and the ChatGPT models described them in greater detail than the HIV.gov Chatbot. Overall, AI-generated information was accurate regarding medication safety and efficacy, yet the study called for improved citation practices and ongoing monitoring of AI updates. | Potential hallucinations where AI provides inaccurate or misleading medication information. Bias toward English-language, western sources. Need for transparent citation of verifiable sources to allow users to assess validity. |
| [25] | Study focuses on three key questions: How do LLM-assisted and human-led analyses differ in their identification and interpretation of themes? What are the relative strengths and limitations of each approach? How might these approaches be effectively combined to enhance qualitative healthcare research? | Constant comparative method and Thematic Analysis | ChatGPT-4o and Gemini Advance Pro 1.5. (Both under a paid subscription) | The LLMs were used to analyze anonymized interview transcripts via a standardized zero-shot prompt to (1) identify main themes related to each patient’s significant experiences and (2) extract illustrative quotes, thereby generating LLM-assisted thematic structures for comparison with investigator-led themes. | **Reliability/ Evaluation Metrics**: Inter-rater reliability (Cohen’s kappa) was calculated only between two human investigators for the manual coding; traditional reliability statistics were not applied to LLM outputs, which were instead assessed via transparent prompts, human review of AI-generated themes and quotes, and conceptual comparison of thematic convergence and divergence between human and AI analyses. | LLMs demonstrated efficiency in identifying recurring patterns but struggled with emotional nuance and contextual depth. Investigator-led analysis, while time-intensive, captured the complexities of identity disruption and emotional processing. Findings suggest that LLMs can serve as complementary tools in qualitative research, enhancing analytical breadth when paired with human interpretation. This study proposes a hybrid model integrating AI-assisted and human-led methods and offers practical recommendations for responsibly incorporating LLMs into qualitative health research. | Ethical considerations included protecting participant privacy in LLM-assisted analysis, ensuring transparency of prompt design, guarding against hallucinated or altered quotes by manually cross-checking all AI-generated excerpts, maintaining human interpretive control over emotionally and identity-laden themes, and adhering to IRB approval and informed consent processes. |
| [26] | This study aimed to determine how well LLM can replicate the human expert evaluation process used in health news quality assessment, which involves providing both a numeric rating and natural language explanations for the ratings. | Qualitative Content Evaluation | ChatGPT-3.5-Turbo | Automated rating of each article on nine HealthNewsReview.org criteria (Cost, Benefit, Harm, Quality, Mongering, Conflict, Alternative, Availability, Novelty) and generation of natural-language explanations accompanying each rating. | **Reliability**: Classification accuracy was compared to the human gold-standard labels using precision, recall, and F1 scores. Inter-rater reliability for the manual explanation assessment exceeded 70%. Consistency and context metrics were independently scored by two reviewers, with a third reviewer resolving disagreements.    **Evaluation Metrics**: Precision, recall, and F1 score for each of the nine criteria. Readability (SMOG score), word count, and sentence count of explanations. Consistency and context quality. | GPT‑3.5‑Turbo showed uneven rating accuracy, strongest for Cost (F1 = 0.824) but weak for other criteria, falling short of supervised ML models. However, it excelled at producing clear, readable, and contextually relevant explanations, highlighting its value for interpretive support despite limited scoring accuracy. Despite limited rating accuracy, GPT‑3.5‑Turbo’s clear and contextually relevant explanations make it valuable for supporting health literacy and helping non‑experts assess health news and misinformation. | The authors note ethical concerns such as the risk of AI-generated misinformation, model bias, and hallucinations; they call for responsible deployment guidelines and monitoring when using LLMs for health communication. |
| [27] | This study aimed to (1) provide detailed guidelines and practices for using ChatGPT in grounded theory within the Chinese context, (2) evaluate the effectiveness of ChatGPT coding by systematically comparing human coding with computer-assisted software to ChatGPT-assisted coding, and (3) explore the broader implications and future directions of ChatGPT in qualitative research. | Grounded theory (open, axial, and selective coding) | ChatGPT-4-Turbo | ChatGPT 4‑Turbo was used to perform open coding, axial coding, and selective coding of the interview transcripts, generating codes, categories, and a theoretical model. | **Reliability**: examined by comparing human‑coded (NVivo) and AI‑coded outputs using semantic alignment, percent agreement, and Cohen’s κ; intercoder reliability for human coding was set > 85% before applying the scheme.  **Evaluation Metrics**: Percent agreement, Cohen’s k, Node and reference-point counts, coverage rate, and t-tests for statistical comparison. | The results revealed that ChatGPT 4-Turbo and manual coding methods exhibited reliability in many aspects. The application of ChatGPT 4-Turbo in grounded theory enhanced the efficiency and diversity of coding and updated the overall grounded theory process. Compared with manual coding, ChatGPT showed shortcomings in depth, context, connections, and coding organization. AI‑assisted coding dramatically reduced analysis time (≈1 day vs. 3 weeks for manual coding). Core categories differed slightly (AI: “impact of gaming on life”; manual: “gaming motivations and impact”), yet the overall theoretical frameworks were largely similar. | Ethics approval was obtained, participants gave informed consent, data were anonymized, and compensation was provided. The authors discuss AI-related risks such as hallucinations, bias, and data-privacy concerns, emphasizing the need for human oversight. |
| [28] | This study explored the capabilities of LLMs ChatGPT-3.5, ChatGPT-4, and Llama 3 to summarize qualitative data from an online brain tumor support forum, assessing the differences between these methods and traditional thematic analysis. | Deductive Thematic Analysis using Dedoose Software for employ a codebook. | ChatGPT-3.5, ChatGPT-4, and Llama 3. | The LLMs were prompted to summarize the content and tone of each of the eight forum posts + responses in a single paragraph, and extract and list the top four challenges expressed by participants. | **Reliability/ Evaluation Metrics**: Assessed by comparing LLM-generated summaries to the human thematic summary using a suite of automatic evaluation metrics (BLEU, ROUGE—1/2/L, METEOR, BERTScore, Cosine and Jaccard similarity) and readability scores (Flesch—Kincaid Grade Level, Reading Ease). Alignment with human themes was also qualitatively examined. | GPT-4 demonstrated superior performance across ROUGE and METEOR metrics, outperforming GPT-3.5 and Llama 3. Semantic similarity scores were comparable across models. GPT-4’s capacity to process entire transcripts increased efficiency, while GPT-3.5 and Llama 3 required data segmenting. Summaries produced by LLMs aligned closely with human-generated thematic analysis, with significant reductions in time and labor. LLMs, particularly GPT-4, show strong potential for summarizing complex, qualitative health data, offering time efficient and consistent outputs. These tools may enhance research efficiency and support in patient-centered environments. However, challenges such as training data biases and capacity limitations in some models warrant further investigation. | The study used only publicly accessible forum posts; usernames and identifying information were removed to ensure anonymity. The IRB at Northern Illinois University exempted the work from human-subjects review. Authors discussed potential bias from LLM training data and capacity limits as ethical considerations for future use. |
| [29] | This study aims to understand how AI-generated health information differs from that provided by the CDC, particularly in terms of sentiment, readability, and overall quality. Language expectancy theory by Burgoon (1995) serves as a framework and offers insights into how people’s expectations of message content from different sources can influence perceived credibility and persuasiveness of such information. | Content Analysis | ChatGPT-3.5 (free version) | The model was prompted to produce health-information responses to 20 open-ended questions about common health topics (e.g., “tell me about high cholesterol”). | **Reliability**: Two coders independently applied the DISCERN tool to both CDC and ChatGPT texts; inter‑rater agreement was assessed with weighted kappa > 0.8, indicating high reliability    **Evaluation Metrics**: The study evaluated the AI-generated health texts using four primary metrics: sentiment analysis, which applied the NRC emotion lexicon via a custom Python script to count anger, sadness, disgust, and other discrete emotions; readability, measured with the Flesch Reading Ease and Flesch-Kincaid Grade Level scores generated through Microsoft Word’s “check accessibility” function; information quality, assessed with the 16-item DISCERN instrument administered by two trained coders; and statistical significance, determined by Wilcoxon signed-rank tests for sentiment differences and independent-samples t-tests for readability and DISCERN scores. | ChatGPT used more negative sentiments, particularly words associated with anger, sadness, and disgust. The CDC’s health messages were significantly easier to read than those generated by ChatGPT. Furthermore, ChatGPT’s responses required a higher reading grade level. In terms of quality, the CDC’s information was a little higher quality than that of ChatGPT, with significant differences in DISCERN scores. Public health professionals need to educate the general public about the complexity and quality of AI-generated health information. Health literacy programs should address topics about quality and readability of AI-generated content. Other recommendations for using AI-generated health information are provided. | The study involved no human participants, animals, or sensitive data and therefore did not require an ethics review. The accuracy of AI responses could be impacted by the timing of the query and its algorithmic attempt to connect it to other events. This can skew the responses toward a certain perspective. AI models also attempt to connect responses to the queries to form a cohesive conversation, which might introduce biases or inaccuracies. AI technologies evolve rapidly, and newer versions may have updates or changes of the underlying algorithms. |
| [30] | The objective of this study was to evaluate to what extent state-of-the-art large language models can appropriately summarize posts shared by patients in web-based forums and health communities. Specifically, the goal was to compare the performance of different language models and prompting strategies on the task of summarizing documents reflecting the experiences of individual patients. | Qualitative Case study (no formal named qualitative analytic framework) | Flan-T5 (flan-t5-base), ChatGPT-3 (text-davinci-003), ChatGPT-3.5 (gpt-3.5-turbo-0301) Note: Open AI API was used. | Abstractive multi-document summarization of patient-authored forum posts grouped into topic-specific sub corpora focused on symptoms and treatments. Systematic comparison of summarization performance under different prompting strategies, including zero-shot, one-shot, three-shot, DSP, CoT, and their combinations. Evaluation of the impact of fine-tuning Flan-T5 on the same summarization task. The GenAI tools were solely used for generating concise summaries of collections of online patient comments about breast cancer symptoms and treatments. | **Reliability**: Human ground truth came from one expert annotator. AI summaries were evaluated against this reference using ROUGE and BERTScore, and differences tested with two‑tailed t tests. With no multiple coders or human rating of outputs, reliability was defined solely as overlap between AI and the single human summary.    **Evaluation Metrics**: ROUGE‑1/2/L measured token‑level overlap, while BERTScore used RoBERTa‑Large embeddings for semantic similarity. Two‑tailed t tests compared mean model and prompt scores. A simple concatenation baseline, created by joining all inputs, was evaluated with the same metrics. | Among the zero-shot prompting–based large language models investigated, GPT-3.5 performed better than the other models with respect to the ROUGE metrics, as well as with respect to BERTScore. While zero-shot prompting seems to be a good prompting strategy, overall GPT-3.5 in combination with directional stimulus prompting in a 3-shot setting had the best results with respect to the metrics. A manual investigation of the summarization of the best-performing method showed that the generated summaries were accurate and plausible compared to the manual summaries. Taken together, results suggest that state-of-the-art pretrained language models are a valuable tool to provide qualitative insights about the patient experience to better understand unmet needs, patient priorities, and how a disease impacts daily functioning and quality of life to inform processes aimed at improving health care delivery and ensure that drug development focuses more on the actual priorities and unmet needs of patients. The key limitations of the work are the small data sample as well as the fact that the manual summaries were created by 1 annotator only. Furthermore, the results hold only for the examined models and prompting strategies, potentially not generalizing to other models and strategies. | The forum posts were automatically anonymized to reduce re‑identification risk and meet GDPR requirements. The authors justified using publicly available posts under GDPR’s legitimate‑interest basis, noting the data are manifestly public and consent is impractical for large, dispersed users. They relied on GDPR provisions allowing processing without explicit consent when obtaining it requires unreasonable effort. Citing ethical guidance for social‑media research, they emphasized anonymization, minimal harm, and public benefit. They also briefly acknowledged general AI risks, including hallucinations and vulnerability to adversarial inputs. |
| [31] | Our objectives for the present study were to (1) identify themes among emergency department leaders’ recommendations for improving telemental health care and (2) compare relative accuracy and efficiency of traditional qualitative analysis versus AI-assisted qualitative analysis. | Inductive codebook development and thematic analysis. | ChatGPT-4 | (1) Generate an initial codebook (theme generation) from all responses; (2) Classify each response into the finalized code categories (coding). | **Reliability**: Coding percentage agreement between AI—augmented and traditional human coders.  **Evaluation Metrics**: a) Percentage agreement; (b) Time spent on codebook development and codebook application. | AI shortened codebook creation (≈ 3 h 30 min vs. 6 h 35 min) and uncovered one novel code missed by human analysts; however, AI‑augmented coding was less accurate (75 % agreement) and more time‑consuming for application, indicating AI’s utility for initial theme generation but limited reliability for final coding without human review. | The study received an IRB exemption, obtained informed consent, and de‑identified responses before AI use. The authors highlight AI’s risk of fabrication and the need for human oversight. Researchers using AI‑augmented analysis became less familiar with the data because immersion and familiarization were bypassed, making it harder to contribute meaningfully to thematic analysis and manuscript development. |
| [32] | This study aims to examine whether 3 ChatGPT chatbots-the World Health Organization’s Sarah, BeFreeGPT, and BasicGPT-provide reliable information on how to quit smoking. | Content Analysis | ChatGPT-4 for building BeFreeGPT and BasicGPT; WHO’s Sarah also built on ChatGPT‑4 technology. | To generate chatbot responses to 12 common quit-smoking queries and to implement the chatbots (BeFreeGPT and BasicGPT) via the ChatGPT “Assistants” API with custom instructions. | **Reliability**: Two independent human coders evaluated each response; disagreements resolved by a third coder, percentage agreement was used.    **Evaluation Metrics**: Overall adherance percentage to the 9-item index; intel-level rates; misinfomation prevalence; engagement; empathy scores; semantic similarity (BERT cosine scores). | Across chatbots and queries, responses met 57.1% of adherence‑index items on average. Sarah performed best (72.2%), significantly outperforming BeFreeGPT (50%) and BasicGPT (47.8%; P<.001). Most responses used clear language (97.3%) and recommended seeking professional counseling (80.3%). About half advised nicotine‑replacement therapy (52.7%), seeking social support (55.6%), or managing cravings (44.4%). Few mentioned non‑nicotine prescription medications (14.1%). Misinformation appeared in 22% of outputs. Challenging queries included quitting “cold turkey,” or quitting with vapes, gummies, a necklace, or hypnosis. All chatbots resisted adversarial jailbreak prompts, refusing or redirecting off‑topic requests. | The study involved no human participants; therefore, it did not require IRB approval. Overall, the study provides support to the idea that LLM chatbots can be designed to adhere to quit-smoking guidelines and counseling principles. While the chatbots we tested responded to some types of queries well, for others, they omitted information, as well as occasionally provided misinformation. |
| [33] | Study aimed to: 1) describe the methodology integrating ChatGPT and human coding in rapid qualitative thematic analysis of LGBTQ+ patients’ positive primary care experiences and how to best improve these experiences, and 2) present the themes derived from this approach. | Deductive Thematic Analysis (Braun & Clarke six‑step: familiarisation, coding, theme searching, reviewing, defining, writing up) | ChatGPT-4 | Automated initial code generation and early‑stage thematic clustering of participants’ free‑text responses; subsequent human review refined codes and defined final themes. | **Reliability:** Human coders cross-checked all ChatGPT outputs, identified “AI hallucinations,” corrected incomplete quotations, and approved or requested re-analysis of code tables.    **Evaluation Metrics:** (1) Frequency of codes and percentage of participants per theme; (2) Cross-reference plots comparing ChatGPT vs. human coding; (3) Correctness flags (yes/no) for each coded instance. | The hybrid approach facilitated the identification of themes related to affirming care practices, provider education, communicative challenges and successes, and environmental cues. While ChatGPT accelerated the coding process, human oversight remained crucial for ensuring data integrity and context accuracy. This hybrid method promises significant improvements in analyzing patient feedback, providing actionable insights that could enhance patient-provider interactions and care for diverse populations. | Institutional Review Board approval; informed consent; participant compensation ($4); discussion of AI-related risks (hallucinations, incomplete quotes) and the necessity of human oversight to safeguard data integrity and participant privacy. |
| [34] | This study aimed to propose new methodologies for enhancing the reliability and efficiency of qualitative research in medicine, as well as to deepen the discussion on the potential of utilizing LLMs for qualitative research. | Inductive thematic analysis following Braun &; Clarke’s six‑step framework | ChatGPT-4 | ChatGPT performed the full thematic analysis workflow (familiarization, code generation, theme searching, theme reviewing, theme definition, final report) on the interview transcripts; it also produced summaries and code lists. | **Reliability:** Three expert assessors (authors/peer reviewers) scored the ChatGPT output on six quality dimensions (depth, confirmability, credibility, transferability, dependability, consistency) using a 1–7 Likert‑type scale, with the human analysis fixed at a reference score of 4.  **Evaluation Metrics:** Quantitative: scores on the six dimensions (depth, confirmability, credibility, transferability, dependability, consistency) ranging from 1–7. Qualitative: free-text comments from assessors describing strengths and weaknesses of the ChatGPT analysis. | ChatGPT achieved higher scores than human analysts on most quality domains, including confirmability, credibility, dependability, and consistency, but showed variable performance on transferability and depth. Assessors reported a strong first impression, clear linkage of data to analysis, and efficient summarization. Limitations included contamination by prompt directions, lack of thick description, and absence of theoretical derivation, leading to shallower contextual insight compared with human analysis. Overall, ChatGPT efficiently extracts key points and generates coherent themes, yet deeper, theory-driven analysis still requires human contextual input. | No formal ethical approval required because only previously published data were used. Authors noted data-privacy considerations of cloud-based AI and the need for careful human verification to avoid prompt contamination and misuse. |
| [35] | This study evaluated the suitability of ChatGPT versions 3.5 and 4 for healthcare professionals seeking up-to-date evidence and recommendations for resuscitation by comparing the key messages of the resuscitation guidelines, which methodically set the gold standard of current evidence and recommendations, with the statements of the AI chatbots on this topic. | Content analysis | ChatGPT-3.5 and the Bing version of ChatGPT-4 (Both free versions) | Prompted to list the key messages of each ERC chapter (BLS, ALS, special circumstances, post-resuscitation care, ethics) and compare AI statements with the official guideline key messages. | **Reliability:** Inter‑rater reliability assessed with Cohen’s κ for completeness and for conformity.    **Evaluation Metrics:** (1) Completeness number/percentage of key messages addressed; (2) Conformity – proportion of AI statements fully in line with the guideline; (3) Error typology (superficial, inaccurate, hallucination, not addressed); (4) Cohen’s κ for rater agreement. | ChatGPT-3.5 generated 60 statements, fully addressing only 13 of 172 key messages; 123 key messages were not addressed. ChatGPT-4 generated 32 statements, fully addressing 20 key messages; 132 key messages were not addressed. Conformity with the ERC text was 77% for 3.5 and 84% for 4. Errors were mainly superficial, inaccurate, or hallucinated; ChatGPT-4 showed higher inter-rater agreement and fewer error types. Authors conclude that clinicians should not rely solely on these chatbots for up-to-date resuscitation knowledge because the guideline content was largely absent from the models’ training data. | This study did not investigate ethical considerations, which are relevant aspects of AI chatbot usage. ERC guidelines are subject to a more general ethical review than ChatGPT and all other LLMs. The origin of the model can impact its ethical performance. Furthermore, all LLMs face the challenge that the volume of training data required exceeds what can be ethically assessed. |
| [36] | Study aimed to assess the feasibility of using LLMs for topic model selection or inductive thematic analysis of large contents of social media posts by attempting to answer the following question: Can LLMs conduct topic model selection and inductive thematic analysis as effectively as humans did in a prior manual study, or at least reasonably, as judged by subject matter experts? | Thematic Analysis (inductive - grounded theory style coding) | GPT‑4 (32 K context), Claude‑Instant (100 K), Claude‑2 (100 K); Note that OpenAI API was used for ChatGPT. | (1) Ranking the 20 bi‑term topics to select the top 5 relevant clusters; (2) Generating five inductive themes with titles, definitions, and exemplar posts from the 768‑tweet corpus. | **Reliability:** The methodology for the two subject‑matter experts who reviewed the LLM‑generated themes, counted matches with the human‑selected top‑5 topics, and assigned “reasonableness” scores (1 = not reasonable, 2 = reasonable, 3 = very reasonable) is detailed in the section on “Assessing Themes in the Responses Given by LLMs”. The procedure for checking the fidelity of the example posts supplied by the LLMs classifying them as identical, near‑identical, or phantom (fabricated) posts is described in the “Assessing Hallucination (Generation of Phantom Posts) in Responses.  **Evaluation Metrics:** Overlap of LLM-ranked top-5 topics with human-selected topics; Number of matched topics; Reasonableness scores; Hallucination rates. | The LLMs consistently ranked topics that humans had previously identified as most relevant, and statistical testing (P<.001) showed they included human‑rated top‑5 content areas far more often than chance. For theme identification, LLMs produced several themes similar to human findings with very low hallucination rates, though results varied across models and repeated runs. Even when themes did not match human outputs exactly, subject matter experts still found them reasonable and relevant. LLMs demonstrated strong ability to process large health‑related social‑media datasets and extract meaningful themes, but they did not reliably reproduce the depth or consistency of human expert analysis. The study highlights substantial future potential for validated LLM‑based real‑time social listening to support public‑health understanding of community concerns and ideas. | The study used de-identified, publicly available Twitter data and received an IRB exemption; authors noted potential bias, hallucinations, and the need for validation before real-time public health deployment. |
| [37] | Study aimed to investigate the potential of LLMs for automating inductive and deductive thematic analysis in qualitative interview studies for the healthcare sector. | Thematic (inductive & deductive) | ChatGPT-3.5-turbo via the API provided by OpenAI, local Mixtral 7x8b model | Two-step prompting: (1) extract themes from each transcript; (2) synthesize and integrate those themes across the entire dataset, effectively performing theme generation and summarization. | **Reliability:** Evaluated by qualitatively comparing the LLM-generated themes to the manually coded “ground-truth” themes to see how closely the AI-derived themes aligned with human annotations.  **Evaluation Metrics:** Qualitative alignment with manual codes, coherence of themes, and ability to capture predefined theoretical constructs (e.g., COM—B); no numeric performance scores were reported. | LLMs produced coherent, theory-aligned themes that matched manual annotations, accelerated thematic analysis, allowed theme extraction even from single interviews (though with some noise), and outperformed Latent Semantic Analysis (topic modelling) which required manual interpretation of word lists; overall, LLMs are a promising, user-friendly complement to existing qualitative automation methods. | Emphasis on careful evaluation for each use-case, avoidance of bias or hallucinated themes, safeguarding privacy by using a local model for unpublished data, and ensuring LLMs do not introduce unsupported findings or propagate harmful stereotypes. |
| [38] | The study evaluated the effectiveness of ChatGPT-4 versus human researchers in qualitative analysis of interviews with patients with adult-acquired buried penis. | Qualitative description approach | Authors used a private ChatGPT‑4 instance, Versa, which operates separately and stores no input data. | Automated coding of interview transcripts, generation of codes, subthemes, and overarching themes for qualitative analysis. | **Reliability:** Assessed via Cohen’s κ coefficient and y analyzing the same interview transcript 10 times with the same prompt set.  **Evaluation Metrics:** Cohen κ for inter‑rater agreement, frequencies/proportions of theme occurrence, agreement matrix, consistency across repeated ChatGPT‑4 runs. | ChatGPT‑4 reliably identified core themes (e.g., body‑image struggles, chronic pain 100% of iterations) with moderate alignment to human coding (κ = 0.401). It captured fewer subthemes and missed some nuances (e.g., fewer “urinary‑spray/stream” and “sexual‑pain” subcodes) but demonstrated consistent theme extraction, supporting a complementary role alongside human analysts. agreement with human analysis. While human analysis provided a richer diversity of subthemes, the consistency of AI suggests its use as a complementary tool in qualitative research. With AI rapidly advancing, future studies should iterate analyses and circumvent token limitations by segmenting data, furthering the breadth and depth of large language model–driven qualitative analyses. | IRB approval was obtained, and the de‑identified secondary analysis was exempted. Researchers used a private GPT‑4 instance (Versa) that stores no input data. The commercialization of LLMs raises concerns that corporate interests may overshadow patient welfare. There is also significant risk of patient harm from incorrect or biased models, underscoring the need for rigorous validation to ensure reliability and avoid adverse clinical outcomes. |
| [39] | The primary aim of the paper was to describe a workflow for producing codes and themes outlined in the Braun and Clarke framework using a locally hosted LLM and advanced prompting strategies. | Thematic analysis following Braun &; Clarke’s six‑step framework | LLaMA-2-70B-Instruct | Study focused on Braun and Clarke’s phases for generating codes, identifying themes, and defining and naming them. These steps align best with LLMs’ strengths in summarization and abstraction and can effectively augment human thematic analysis by combining machine efficiency with human interpretive insight. | **Reliability:** Three evaluation methods: (1) hand-coding by three independent human raters; Sentence-T5-xxl cosine-similarity with threshold conversion; LLM-based binary overlap prompt.    **Evaluation Metrics:** Jaccard similarity coefficients; cosine similarity scores; and inter-rater reliability. | These revealed similarities ranging from moderate to substantial (Jaccard similarity coefficients 0.44–0.69), which are promising preliminary results. Authors felt that this study is a major step in validating the idea that open-source LLMs are or could be powerful tools for TA, extending this class of tools even into projects where Protected Health information is involved. This study demonstrates that open-source LLMs can effectively generate robust themes from qualitative data, achieving substantial similarity to human-generated themes. The validation of LLMs in thematic analysis, coupled with evaluation methodologies, highlights their potential to enhance and democratize qualitative research across diverse fields. | This study was deemed exempt from IRB review by the Yale University Human Research Protection Program. All processing performed locally to avoid PHI exposure and comply with HIPAA/GDPR; discussion of embedded bias in LLMs and need for bias mitigation; acknowledgement that commercial LLM services lack Business Associate Agreements for protected health information. |
| [40] | The aim of this study was to explore the utility of ChatGPT in conducting qualitative content analysis through the analysis of forum posts from people sharing their experiences on reducing their sugar consumption. | Content Analysis (inductive & deductive, theoretical domains framework was used) | ChatGPT-3.5-Turbo (free version) | Extraction of “change mechanisms” from forum posts (data‑preparation); 2) Development of 10 inductive, data‑driven coding schemes; 3) Development of 10 unconstrained deductive, TDF‑based coding schemes; 4) Direct coding of the full dataset into a structured TDF coding matrix. | **Reliability/ Evaluation Metrics:** Compared ChatGPT output to human coding using (a) precision of mechanism extraction; (b) intercoder agreement (Fleiss κ) for each coding scheme. | Precision for detecting change mechanisms ranged from 66% to 88%. Intercoder agreement showed κ scores of 0.72–0.82 for inductive schemes and 0.58–0.73 for unconstrained and structured schemes. The best-performing scheme produced κ values of 0.67–0.95 (inductive) and 0.13–0.87 (deductive). ChatGPT generally followed prompt instructions, though inductive scheme descriptions were longer than requested. It proved reliable in assisting qualitative analysis, performing better when generating inductive codes from data than when adapting or applying existing frameworks. ChatGPT also showed promise as a second coder, achieving near‑perfect agreement in one scheme. Overall, it may support all phases of qualitative content analysis, though multiple iterations are needed to confirm reliability. | Data were open-access and exempt from ethics review; authors highlighted the need for transparency, informed consent, data-privacy safeguards, and awareness of potential biases in LLM training data. |
| [41] | This study explores the potential of using large language models to assist content analysis by conducting a case study to identify adverse events (AEs) in social media posts. The case study compares ChatGPT’s performance with human annotators’ in detecting AEs associated with delta-8-tetrahydrocannabinol, a cannabis-derived product. | Content Analysis | ChatGPT-3.5-Turbo (OpenAI API used) | Automated annotation of 10,000 Reddit posts for the presence of any adverse event and for seriousness according to FDA MedWatch categories, using the same codebook given to human annotators. | **Reliability/ Evaluation Metrics:** Assessed via absolute and prevalence‑adjusted Fleiss κ agreement between ChatGPT outputs and human annotations. | Using the identical instructions given to human annotators, ChatGPT closely approximated human results, with a high degree of agreement noted: 94.4% (9436/10,000) for any AE detection (Fleiss κ=0.95) and 99.3% (9931/10,000) for serious AEs (κ=0.96). These findings suggest that ChatGPT has the potential to replicate human annotation accurately and efficiently. The study recognizes possible limitations, including concerns about the generalizability due to ChatGPT’s training data, and prompts further research with different models, data sources, and content analysis tasks. The study highlights the promise of large language models for enhancing the efficiency of biomedical research. | Study exempt from IRB because data were public and non-identifiable; authors note potential training-data overlap that could inflate performance and discuss the need for transparency and broader validation when applying LLMs to biomedical text. |
| [42] | This study aims to examine ChatGPT’s capability to debunk sleep-related disbeliefs. | Qualitative comparative analysis | ChatGPT-4 | ChatGPT categorized 20 sleep-related myths identified by 10 sleep experts and rated them in terms of falseness and public health significance, on a 5-point Likert scale. | **Reliability:** Inter‑rater reliability assessed via intraclass correlation coefficients (ICCs) between ChatGPT’s ratings and expert ratings.  **Evaluation Metrics:** Sensitivity, Positive Predictive Value, Intra class correlation, mean likert scores for falseness and for public health significance. | ChatGPT labeled 85% of statements as false or generally false, with accuracy varying by topic. It performed well on myths about sleep timing, duration, and behaviors during sleep, but showed mixed accuracy for pre‑sleep behaviors and brain function. Its Likert‑scale ratings of falseness (mean 3.45) and public‑health significance (mean 3.15) showed good accuracy. The tool demonstrated 85% sensitivity and a 100% positive predictive value, meaning its false labels were highly reliable, though some false statements were missed. ChatGPT’s appraisals aligned closely with expert judgments, with high ICCs for falseness (.83) and public‑health impact (.79). Qualitatively, both ChatGPT and experts rejected sleep myths, but ChatGPT used more accessible, generalized explanations, whereas experts provided more technical, evidence‑based detail. | ChatGPT cannot completely replace expert opinions, especially in nuanced and complex fields such as sleep health, but can be a valuable complement in the dissemination of updated information and promotion of healthy behaviors. |
| **Important Abbreviations in the Table**: IRB = Internal Review Board; HIV = Human Immunodeficiency Virus; PrEP = Pre-Exposure Prophylaxis; AI = Artificial Intelligence; SMS = Short Message Service; LLMs = Large Language Models; ICR = Inter-coder reliability; GenAI = Generative Artificial Intelligence; CLD = Causal Loop Diagram; GDPR = General Data Protection Regulation; QCA = Qualitative Content Analysis; LDA = Latent Dirichlet Allocation; CVD = cardiovascular disease; CV = Cardiovascular; HIPPA = Health Insurance Portability and Accountability Act; SES = Socio-economic status; API = Application Programming Interface; RTA = Reflexive Thematic Analysis; SOC 2 = Systems and Organization Controls; QBA = Query-Based Analysis; CAQDAS = Computer-assisted qualitative data analysis software; SMOG = simple Measure of Gobbledygook; NRC = National Research Council; DSP = Directional Stimulus Prompting; CoT = Chain of Thought Prompting; BLS = Basic Life Support; ALS = Advanced Life Support; ERC = European Resuscitation Council Guidelines; COM-B = Capability, Opportunity, Motivation – Behavior. | | | | | | | |
| **Important References in the Table:** Feldman, L., Hart, P. S., & Milosevic, T. (2017). Polarizing news? Representations of threat and efficacy in leading US newspapers' coverage of climate change. Public understanding of science (Bristol, England), 26(4), 481–497. <https://doi-org.libproxy.washu.edu/10.1177/0963662515595348>; Landis, J. R., & Koch, G. G. (1977). The measurement of observer agreement for categorical data. Biometrics, 33(1), 159–174.; Methodological challenges in qualitative content analysis: a discussion paper. Graneheim UH, Lindgren BM, Lundman B. Nurse Educ Today. 2017;56:29–34. doi: 10.1016/j.nedt.2017.06.002; Qualitative content analysis in nursing research: concepts, procedures and measures to achieve trustworthiness. Graneheim UH, Lundman B. Nurse Educ Today. 2004;24:105–112. doi: 10.1016/j.nedt.2003.10.001.; Donabedian A. (1966). Evaluating the quality of medical care. Milbank Memorial Fund Quarterly, 44(3), 166–206.; Burgoon M. Language expectancy theory: elaboration, explication, and extension. In: CR Berger, M Burgoon, editor. Communication and social influence processes.East Lansing, MI: Michigan State University Press; 1995. p. 29–52. [43-47] | | | | | | | |

**References Appendix**

1. Han Z, Tavasi A, Lee JY, Luzuriaga J, Suresh K, Oppenheim M, et al. Can large language models be used to code text for thematic analysis? An explorative study. Discover Artificial Intelligence. 2025;5:171. doi:10.1007/s44163-025-00441-3.
2. Wachinger J, Bärnighausen K, Schäfer LN, et al. Prompts, pearls, imperfections: Comparing ChatGPT and a human researcher in qualitative data analysis. Qual Health Res. 2025;35(9):951‑966. doi:10.1177/10497323241244669.
3. Chandler RD, Warner S, Aidoo‑Frimpong G, Wells J. What did you say, ChatGPT? The use of AI in Black women’s HIV self‑education: An inductive qualitative data analysis. J Assoc Nurses AIDS Care. 2024;35(3):294‑301.
4. Ferguson C. A researcher’s journey to the use of AI for qualitative data analysis: Findings from a test case with ChatGPT. Issues Educ Res. 2025;35(1):126‑141. <http://www.iier.org.au/iier35/ferguson.pdf>
5. Farjam M, Meyer H, Lohkamp M. A practical guide and case study on how to instruct LLMs for automated coding during content analysis. Soc Sci Comput Rev. 2025. doi:10.1177/08944393251349541.
6. Prescott MR, Yeager S, Ham L, et al. Comparing the Efficacy and Efficiency of Human and Generative AI: Qualitative Thematic Analyses. JMIR AI. 2024;3:e54482. Published 2024 Aug 2. doi:10.2196/54482
7. Qiao S, Fang X, Wang J, Zhang R, Li X, Kang Y. Generative AI for thematic analysis in a maternal health study: coding semistructured interviews using large language models. Applied Psych Health &amp; Well. 2025;17(3):e70038. doi:10.1111/aphw.70038
8. Jalali MS, Akhavan A. Integrating AI language models in qualitative research: Replicating interview data analysis with ChatGPT. Syst Dyn Rev. 2024;40(3):e1772. doi:10.1002/sdr.1772.
9. Sakaguchi K, Sakama R, Watari T. Evaluating ChatGPT in Qualitative Thematic Analysis With Human Researchers in the Japanese Clinical Context and Its Cultural Interpretation Challenges: Comparative Qualitative Study. J Med Internet Res. 2025;27:e71521. Published 2025 Apr 24. doi:10.2196/71521
10. Balt E, Salmi S, Bhulai S, Vrinzen S, Eikelenboom M, Gilissen R, et al. Deductively coding psychosocial autopsy interview data using a few‑shot learning large language model. Front Public Health. 2025;13:1512537. doi:10.3389/fpubh.2025.1512537.
11. Lockwood A, Newman DS, Mossing KW, Glubzinski A, Cohen E. Human versus machine: A comparative analysis of qualitative coding by humans and ChatGPT‑4. Sch Psychol. Published online October 20, 2025. doi:10.1037/spq0000715.
12. Castellanos A, Jiang H, Gomes P, Vander Meer D, Castillo A. Large language models for thematic summarization in qualitative healthcare research: Comparative analysis of model and human performance. JMIR AI. 2025;4:e64447. doi:10.2196/64447.
13. Kon MHA, Pereira MJ, De Castro Molina JAD, Yip VCH, Abisheganaden J, Yip WF. Unravelling ChatGPT’s potential in summarising qualitative in‑depth interviews. Eye. 2025;39:354–358. doi:10.1038/s41433-024-03419-0.
14. Lund‑Tonnesen M, Vahr Lauridsen S, Rosenberg J. Evaluating Microsoft Copilot in qualitative health research: Accurate for manifest content coding but limited in latent interpretation. Cureus. 2025;17(10):e95719. doi:10.7759/cureus.95719.
15. Goldberg EM, Macis M, Bounds M, Picazo JG, Nicholas LH. Free‑text responses in a nationally representative experimental survey about end‑of‑life care choices: ChatGPT‑4o‑assisted qualitative analytical study. JMIR Aging. 2025;8:e76335. doi:10.2196/76335.
16. Kermansaravi M, Cohen RV. Clinical obesity through the lens of context‑aware large language models. Obes Surg. 2025;35:5247‑5255. doi:10.1007/s11695-025-08341-2.
17. Chubb LA, Jackson S, Naseer B, Matthews M. To leave or stay? Influences on early exit and completion in a New Zealand residential drug rehabilitation service. Qual Health Res. 2025;0(0):1‑16. doi:10.1177/10497323251367177.
18. Parameswaran V, Bernard J, Bernard A, Deo N, Tsung S, Lyytinen K, et al. Evaluating large language models and retrieval‑augmented generation enhancement for delivering guideline‑adherent nutrition information for cardiovascular disease prevention: Cross‑sectional study. J Med Internet Res. 2025;27:e78625. doi:10.2196/78625.
19. Hairston JM, Ranjan R, Lakamana S, Spadaro A, Bozkurt S, Perrone J, Sarker A. Automating inductive thematic analyses of health content using large language models: A proof‑of‑concept study using social media data. JAMIA Open. 2025;8(5):ooaf102. doi:10.1093/jamiaopen/ooaf102.
20. Vikan M, Aryan R, Kannelønning MS, Riegler D, Danielsen S, Alexander SO. Reflecting on LLM support in reflexive thematic analysis: An exploratory study. Qual Health Res. 2025;0(0):1‑15. doi:10.1177/10497323251365211.
21. Jeminiwa RN, Popielaski C, King A. Exploring young adults' experiences and beliefs in asthma medication management: Pilot qualitative study comparing human and multiple AI thematic analyses. JMIR Form Res. 2025;9:e69892. doi:10.2196/69892.
22. Misgav K, Neufeld‑Kroszynski G, Palombo M, Karnieli‑Miller O. Human analysis vs. artificial intelligence: Analyzing qualitative medical students’ narratives. Qual Health Res. 2025;0(0):1‑13. doi:10.1177/10497323251359445.
23. Morgan DL. Query‑based analysis: A strategy for analyzing qualitative data using ChatGPT. Qual Health Res. 2025. doi:10.1177/10497323251321712.
24. Beegle S, Gomez LA, Blackard JT, et al. HIV prevention and treatment information from four artificial intelligence platforms: A thematic analysis. AIDS Behav. 2025;29(11):3394‑3403. doi:10.1007/s10461-025-04786-9.
25. Shanwetter Levit N, Saban M. When investigator meets large language models: A qualitative analysis of cancer patient decision‑making journeys. npj Digit Med. 2025;8:336. doi:10.1038/s41746-025-01747-3.
26. Liu X, He L, Alanazi E, et al. Assessing the accuracy and explainability of using ChatGPT to evaluate the quality of health news. BMC Public Health. 2025;25:2038. doi:10.1186/s12889-025-23206-0.
27. Yue Y, Liu D, Lv Y, Hao J, Cui P. A practical guide and assessment on using ChatGPT to conduct grounded theory: Tutorial. J Med Internet Res. 2025;27:e70122. doi:10.2196/70122.
28. Muasher‑Kerwin C, Hughes MC, Foster ML, Al Azher I, Alhoori H. Exploring large language models for summarizing and interpreting an online brain tumor support forum. Digit Health. 2025;11:20552076251337345. doi:10.1177/20552076251337345.
29. Young A, Omosun F. A comparative analysis of CDC and AI‑generated health information using computer‑aided text analysis. J Commun Healthc. 2025;3:205‑216. doi:10.1080/17538068.2025.2487378.
30. Nair RAS, Hartung M, Heinisch P, et al. Summarizing online patient conversations using generative language models: Experimental and comparative study. JMIR Med Inform. 2025;13:e62909. doi:10.2196/62909.
31. Keating C, Marcus SC, Bowden CF, Worsley D, Doupnik SK. Artificial intelligence and qualitative analysis of emergency department telemental health care implementation survey. Telemed J E Health. 2025;31(7):821‑828. doi:10.1089/tmj.2024.0555.
32. Abroms LC, Yousefi A, Wysota CN, Wu T‑C, Broniatowski DA. Assessing the adherence of ChatGPT chatbots to public health guidelines for smoking cessation: Content analysis. J Med Internet Res. 2025;27:e66896. doi:10.2196/66896.
33. Stage MA, Creamer MM, Ruben MA. Having providers who are trained and have empathy is life‑saving: Improving primary‑care communication through thematic analysis with ChatGPT and human expertise. PEC Innov. 2024;6:100371. doi:10.1016/j.pecinn.2024.100371.
34. Kondo T, Miyachi J, Jönsson A, Nishigori H. A mixed‑methods study comparing human‑led and ChatGPT‑driven qualitative analysis in medical education research. Nagoya J Med Sci. 2024;86(4):620‑644. doi:10.18999/nagjms.86.4.620.
35. Beck S, Kuhner M, Haar M, et al. Evaluating the accuracy and reliability of AI chatbots in disseminating the content of current resuscitation guidelines: A comparative analysis. Scand J Trauma Resusc Emerg Med. 2024;32:95. doi:10.1186/s13049‑024‑01266‑2.
36. Deiner MS, Honcharov V, Li J, Mackey TK, Porco TC, Sarkar U. Large language models can enable inductive thematic analysis of a social media corpus in a single prompt: Human validation study. JMIR Infodemiology. 2024;4:e59641. doi:10.2196/59641.
37. Wosny M, Hastings J. Applying large language models to interpret qualitative interviews in healthcare. Stud Health Technol Inform. 2024;316:791‑795. doi:10.3233/SHTI240530.
38. Li KD, Fernandez AM, Schwartz R, Rios N, Carlisle MN, Amend GM, et al. Comparing GPT‑4 and human researchers in health care data analysis: Qualitative description study. J Med Internet Res. 2024;26:e56500. doi:10.2196/56500.
39. Mathis WS, Zhao S, Pratt N, Weleff J, De Paoli S. Inductive thematic analysis of healthcare qualitative interviews using open‑source LLMs: How does it compare to traditional methods? Comput Methods Programs Biomed. 2024;255:108356. doi:10.1016/j.cmpb.2024.108356.
40. Bijker R, Merkouris SS, Dowling NA, Rodda SN. ChatGPT for automated qualitative research: Content analysis. J Med Internet Res. 2024;26:e59050. doi:10.2196/59050.
41. Leas EC, Ayers JW, Desai N, Dredze M, Hogarth M, Smith DM. Using large language models to support content analysis: A case study of ChatGPT for adverse event detection. J Med Internet Res. 2024;26:e52499. doi:10.2196/52499.
42. Bragazzi NL, Garbarino S. Assessing the accuracy of generative conversational artificial intelligence in debunking sleep health myths: Mixed‑methods comparative study with expert analysis. JMIR Form Res. 2024;8:e55762.
43. Feldman L, Hart PS, Milosevic T. Polarizing news? Representations of threat and efficacy in leading US newspapers' coverage of climate change. Public Underst Sci. 2017;26(4):481-497. doi:10.1177/0963662515595348
44. Landis JR, Koch GG. The measurement of observer agreement for categorical data. Biometrics. 1977;33(1):159-174.
45. Graneheim UH, Lundman B. Qualitative content analysis in nursing research: concepts, procedures and measures to achieve trustworthiness. Nurse Educ Today. 2004;24(2):105-112. doi:10.1016/j.nedt.2003.10.001
46. Donabedian A. Evaluating the quality of medical care. 1966. Milbank Q. 2005;83(4):691-729. doi:10.1111/j.1468-0009.2005.00397.x
47. Burgoon, M. (1995). Language expectancy theory: Elaboration, explication, and extension. InC. R. Berger & M. Burgoon (Eds.), Communication and social influence processes (pp. 29–52).East Lansing, MI: Michigan State University Press.
