# Supplementary material for: Generative Artificial Intelligence for Qualitative Methods in Health Research: Rapid Review
Source: J Med Internet Res. 2026 Aug 31;28:e98551. doi: 10.2196/98551 (PMC13536981; doi:10.2196/98551)
Supplement: Supplementary file 6 [file jmir-v28-e98551-s006.docx]

Supplementary Appendix V

| **Appendix V.** CASP Checklist | | | | | | | | | | | |
| --- | --- | --- | --- | --- | --- | --- | --- | --- | --- | --- | --- |
| **Study** | Research Aims | Methodological Fit | Research Design | Approach to inquiry | Recruitment strategy | Data collection | Reflexivity | Ethics | Data analysis rigor | Clear Findings | Value of the Research |
| [1] | Y | Y | Y | S | Y | Y | CT | Y | Y | Y | The authors conclude that LLMs can speed initial coding but require major improvements, offering actionable insights for future methodological development. |
| [2] | Y | Y | Y | Y | Y | Y | Y | Y | S | Y | Provides empirical evidence on AI‑assisted qualitative analysis, highlights practical opportunities (speed, triangulation) and risks (fabrication, lack of reflexivity), and outlines future research directions. |
| [3] | Y | Y | Y | S | Y | Y | S | S | S | Y | The study advances understanding of algorithmic bias, explores ChatGPT for sexual health education, and offers practical guidance for equity-focused AI implementation with recommendations for cautious use and standardized evaluation. |
| [4] | Y | Y | Y | Y | Y | Y | Y | Y | S | Y | The study contributes practical guidance for researchers using generative AI (e.g., need for manual analysis first, specific prompts, expert knowledge). The sample is narrow, limiting transferability, but the methodological insight and ethical discussion are valuable for emerging AI‑assisted qualitative work. |
| [5] | Y | Y | Y | Y | Y | Y | Y | Y | Y | Y | Demonstrates practical LLM integration for systematic qualitative content analysis, offering a scalable, multilingual, cost‑effective alternative to manual coding. The generalizable workflow supports large samples, improves transparency and replicability, while acknowledging limits of LLM understanding and need for human oversight. |
| [6] | Y | Y | Y | Y | Y | Y | S | Y | S | Y | The study is the first to compare ChatGPT and Bard using inductive and deductive health data analysis, offering timely guidance on hybrid human-AI qualitative workflows, while noting limits related to SMS data, non-naturalistic sources, and domain-specific focus. |
| [7] | Y | Y | Y | S | S | Y | N | Y | S | Y | Transferability is limited by the small, single‑state sample, use of one GenAI model, and minimal reflexive thematic interpretation. Nonetheless, the study offers a valuable proof‑of‑concept and early benchmark for public health researchers integrating generative AI into thematic analysis workflows. |
| [8] | Y | Y | S | S | Y | S | S | S | S | Y | The study demonstrates that generative AI can augment qualitative health research by expanding CLD depth while highlighting methodological, ethical, and reproducibility challenges. It offers a practical example and balanced critique, making a meaningful methodological contribution to the field. |
| [9] | Y | Y | Y | S | Y | Y | Y | Y | Y | Y | The study offers an early examination of ChatGPT in non‑English qualitative health research, showing strong performance for descriptive themes but limitations with culturally nuanced Japanese concepts. It underscores the need for human interpretation, supports hybrid AI–human methods, and notes limited transferability due to the small, exploratory, single‑country sample. |
| [10] | Y | Y | Y | S | Y | Y | CT | Y | Y | Y | An LLM significantly accelerates deductive coding of psychosocial‑autopsy interviews with acceptable reliability. The study transparently notes risks (false positives, hallucinations) and advocates collaborative human–AI workflows, offering methodological guidance for suicide‑prevention research. |
| [11] | Y | Y | Y | S | S | S | Y | Y | Y | Y | The study offers one a side‑by‑side empirical comparisons of human coders with ChatGPT‑4, showing AI’s speed advantage but limited interpretive depth, and provides practical guidance for training, ethical oversight, and AI development in qualitative research. |
| [12] | Y | Y | Y | S | S | Y | N | Y | Y | Y | The study shows LLMs can reliably augment thematic interpretation while identifying key limitations such as bias, over‑fitting, and need for researcher oversight. Its methodological contribution is strong, though generalizability is limited by use of a single forum and LLM family. |
| [13] | Y | Y | Y | S | Y | Y | N | Y | Y | Y | This research is highly valuable for qualitative health researchers and practitioners. It provides empirical evidence that ChatGPT can significantly accelerate rapid preliminary analysis while emphasizing that it remains a collaborative tool that still requires human oversight to ensure nuance and ethical integrity. |
| [14] | Y | Y | Y | Y | Y | Y | S | Y | Y | Y | This research offers practical guidance for integrating AI into qualitative workflows, showing where AI can reduce workload while emphasizing the continued necessity of human oversight for interpretive depth, and contributing meaningfully to AI‑augmented qualitative methodology. |
| [15] | Y | Y | Y | S | Y | Y | Y | Y | Y | Y | The study is highly valuable clinically and methodologically. It shows surrogates’ end‑of‑life decisions often rely on personal experience rather than patient wishes, underscoring gaps in advance care planning, while offering a pioneering roadmap for efficient LLM‑assisted qualitative analysis with essential human validation. |
| [16] | Y | Y | Y | Y | Y | Y | S | Y | S | Y | The research is highly valuable as it establishes a practical five-step model for integrating collaborative intelligence into medical guideline development. It demonstrates that AI can significantly accelerate knowledge synthesis while highlighting critical risks, such as hallucinated citations, that require ongoing expert vigilance. |
| [17] | Y | Y | Y | Y | Y | Y | Y | Y | Y | Y | This research is highly valuable as it offers both methodological innovation (AI-assisted qualitative analysis) and practical insights for addiction services. It suggests specific policy reforms, such as adopting trauma-and-violence-informed (TVI) methods and increasing family inclusion to improve treatment retention. |
| [18] | Y | Y | Y | Y | Y | Y | CT | Y | Y | Y | The research provides critical insights into the safety risks of off-the-shelf LLMs (which generated harmful content) and demonstrates how RAG integration can ground AI responses in evidence-based guidelines. It offers a practical framework for healthcare institutions considering the deployment of LLMs for clinical nutrition guidance. |
| [19] | Y | Y | Y | S | Y | Y | N | Y | Y | Y | This study shows LLMs can accurately replicate expert thematic labels, offering a scalable, cost‑ and time‑efficient supplement to manual qualitative analysis. It enables near–real-time drug surveillance from social media, but still requires human experts to define and initiate analytic themes. |
| [20] | Y | Y | Y | Y | Y | Y | Y | Y | Y | Y | This research is highly valuable for the qualitative community. It provides a critical reality check on the hype surrounding AI, demonstrating that LLMs currently lack the reflexive depth required for high-quality RTA. It offers a practical framework (prompt strategy) while warning of significant risks, such as data fabrication and epistemological misalignment. |
| [21] | Y | Y | Y | Y | S | Y | Y | Y | S | Y | This pilot study shows AI can rapidly perform thematic analysis with high agreement to human coders, offering a framework for larger studies. It highlights digital health features for asthma care, though findings have limited generalizability due to a small, specific sample. |
| [22] | Y | Y | Y | Y | Y | Y | Y | Y | Y | Y | This study proposes a hybrid framework for scalable analysis of student narratives to inform medical education and systemic insights, while demonstrating LLM effectiveness in Hebrew and other non‑English contexts. |
| [23] | Y | Y | Y | Y | Y | Y | Y | Y | Y | Y | The research provides a systematic, time-saving method for qualitative researchers to leverage generative AI. It contributes to the evolving methodology of qualitative health research by offering a framework that maintains a "human in the loop" while enhancing analytic efficiency. |
| [24] | Y | Y | Y | S | Y | Y | S | CT | Y | Y | This exploratory study systematically evaluates accessible AI tools for delivering sensitive health information. Despite rapid model evolution limiting durability of findings, its longitudinal design and rigorous coding offer important insights into LLM reliability and risks for vulnerable populations. |
| [25] | Y | Y | Y | S | Y | Y | S | Y | Y | Y | This study fills a key gap by showing LLMs capture structural patterns but miss emotional and identity nuances. It offers practical “dos and don’ts” for hybrid AI-human qualitative research, reinforcing the irreplaceable role of human interpretation in patient experience. |
| [26] | Y | Y | Y | S | Y | Y | CT | Y | Y | Y | This study highlights a key gap: LLMs generate convincing explanations but lack accuracy in health news evaluation. It offers actionable guidance for hybrid systems combining supervised learning with LLM explanations to strengthen public health literacy. |
| [27] | Y | Y | Y | Y | S | Y | S | Y | Y | Y | This study shows AI can dramatically accelerate qualitative coding, from weeks to a single day, while offering a practical human‑AI collaboration framework. It emphasizes that human expertise remains essential to ensure rigor and address algorithmic bias. |
| [28] | Y | Y | Y | S | Y | Y | Y | Y | Y | Y | The study shows that GPT‑4 can dramatically reduce qualitative analysis time while producing results comparable to human analysts. It emphasizes efficiency gains alongside current limitations, noting humans are better at identifying complex emotions and unmet needs, offering a balanced view of AI’s role in qualitative health research. |
| [29] | Y | Y | Y | Y | Y | Y | CT | Y | Y | Y | The study shows AI health messages are often less readable and emotionally colder than official sources, posing risks for health literacy and disparities. It underscores the need for public education on the limitations of AI-generated health information. |
| [30] | Y | Y | Y | S | Y | Y | S | Y | Y | Y | The study offers a scalable way to extract patient experience insights from large datasets, showing LLMs can accurately summarize unmet needs and quality‑of‑life impacts while accelerating drug development, trial design, and care optimization, and providing a key benchmark dataset for future research. |
| [31] | Y | Y | Y | S | Y | Y | Y | Y | Y | Y | The study shows that LLMs like GPT‑4 can dramatically reduce qualitative analysis time while producing results comparable to humans. Although faster and unaffected by fatigue, LLMs still lag slightly in identifying complex emotions and unmet needs, underscoring the value of human-AI collaboration. |
| [32] | Y | Y | Y | S | Y | Y | S | Y | Y | Y | This research benefits both medical and NLP fields by showing how LLMs can extract qualitative patient insights at scale. It demonstrates accurate summarization of unmet needs and quality‑of‑life impacts, supports faster drug and trial development, and introduces a benchmark dataset for future health informatics research. |
| [33] | Y | Y | Y | Y | Y | Y | CT | Y | Y | Y | This study is a valuable first evaluation of LLM chatbots for smoking cessation support, introducing a reproducible adherence index and revealing that even specialized bots can deliver misinformation. It underscores the need for strong guardrails and establishes a safety‑first baseline for public health deployment. |
| [34] | Y | Y | Y | Y | Y | Y | Y | Y | Y | Y | This research is highly valuable as it introduces a novel hybrid methodology for rapid qualitative analysis in healthcare. It provides actionable insights (e.g., specific training protocols and environmental cues) that can be directly applied to improve patient-provider communication for LGBTQ+ individuals. |
| [35] | Y | Y | Y | Y | Y | Y | Y | Y | Y | Y | This research offers evidence‑based caution for healthcare professionals and educators, showing that current AI chatbots lack deep conceptual understanding and may propagate clinical misconceptions. It highlights risks in life‑critical contexts while proposing practical enhancements, such as retrieval‑augmented generation, to improve AI reliability in medical communication. |
| [36] | Y | Y | Y | Y | Y | Y | S | Y | Y | Y | This study advances infodemiology by demonstrating major efficiency gains for automated social listening, cutting analysis from over 40 hours to a few. It identifies a realistic starting point for hybrid human–LLM monitoring while transparently outlining current limitations in nuance and reliability. |
| [37] | Y | Y | Y | S | S | Y | CT | S | Y | Y | This exploratory study serves as a proof of concept for accelerating qualitative healthcare research, showing that LLMs capture richer theoretical nuance than traditional topic models while reducing time and costs. It highlights potential for broader stakeholder inclusion, while noting the need to address bias and ensure interpretive accuracy. |
| [38] | Y | Y | Y | Y | Y | Y | S | Y | Y | Y | This research offers a practical framework for integrating AI into qualitative health research, showing GPT‑4 as a complementary tool that accelerates coding while balancing strengths like speed and consistency against limits in depth, guiding realistic human-AI collaboration in academic and medical contexts. |
| [39] | Y | Y | Y | Y | Y | Y | S | Y | Y | Y | This research advances qualitative healthcare methods by validating open‑source LLMs for thematic analysis of sensitive data, showing strong alignment with human experts while reducing cognitive and resource burdens. It highlights AI’s potential to democratize research while emphasizing the continued necessity of human oversight. |
| [40] | Y | Y | Y | Y | Y | Y | Y | Y | Y | Y | This study is valuable as an early systematic test of ChatGPT for complex qualitative research, establishing a framework for AI‑assisted qualitative analysis while showing that AI reduces workload but still requires human oversight to ensure rigor, alignment, and replicability across domains. |
| [41] | Y | Y | Y | S | Y | Y | CT | Y | Y | Y | The research serves as a proof of concept for LLMs in biomedical research, showing major time and cost savings and near‑perfect replication of human‑identified adverse events in unstructured data, while noting the need for further validation across datasets and more complex annotation tasks. |
| [42] | Y | Y | Y | S | Y | Y | CT | Y | Y | Y | This research fills a critical gap in understanding generative AI and medical misinformation, showing ChatGPT as a useful complement to experts but limited in nuanced fields like public and occupational health, while offering a transferable framework to assess and improve AI accuracy. |
| **Note**: “Y” = Yes; “N” = No; “CT” = Can’t Tell; “S” = Somewhat | | | | | | | | | | | |

**References Appendix**

1. Han Z, Tavasi A, Lee JY, Luzuriaga J, Suresh K, Oppenheim M, et al. Can large language models be used to code text for thematic analysis? An explorative study. Discover Artificial Intelligence. 2025;5:171. doi:10.1007/s44163-025-00441-3.
2. Wachinger J, Bärnighausen K, Schäfer LN, et al. Prompts, pearls, imperfections: Comparing ChatGPT and a human researcher in qualitative data analysis. Qual Health Res. 2025;35(9):951‑966. doi:10.1177/10497323241244669.
3. Chandler RD, Warner S, Aidoo‑Frimpong G, Wells J. What did you say, ChatGPT? The use of AI in Black women’s HIV self‑education: An inductive qualitative data analysis. J Assoc Nurses AIDS Care. 2024;35(3):294‑301.
4. Ferguson C. A researcher’s journey to the use of AI for qualitative data analysis: Findings from a test case with ChatGPT. Issues Educ Res. 2025;35(1):126‑141. <http://www.iier.org.au/iier35/ferguson.pdf>
5. Farjam M, Meyer H, Lohkamp M. A practical guide and case study on how to instruct LLMs for automated coding during content analysis. Soc Sci Comput Rev. 2025. doi:10.1177/08944393251349541.
6. Prescott MR, Yeager S, Ham L, et al. Comparing the Efficacy and Efficiency of Human and Generative AI: Qualitative Thematic Analyses. JMIR AI. 2024;3:e54482. Published 2024 Aug 2. doi:10.2196/54482
7. Qiao S, Fang X, Wang J, Zhang R, Li X, Kang Y. Generative AI for thematic analysis in a maternal health study: coding semistructured interviews using large language models. Applied Psych Health &amp; Well. 2025;17(3):e70038. doi:10.1111/aphw.70038
8. Jalali MS, Akhavan A. Integrating AI language models in qualitative research: Replicating interview data analysis with ChatGPT. Syst Dyn Rev. 2024;40(3):e1772. doi:10.1002/sdr.1772.
9. Sakaguchi K, Sakama R, Watari T. Evaluating ChatGPT in Qualitative Thematic Analysis With Human Researchers in the Japanese Clinical Context and Its Cultural Interpretation Challenges: Comparative Qualitative Study. J Med Internet Res. 2025;27:e71521. Published 2025 Apr 24. doi:10.2196/71521
10. Balt E, Salmi S, Bhulai S, Vrinzen S, Eikelenboom M, Gilissen R, et al. Deductively coding psychosocial autopsy interview data using a few‑shot learning large language model. Front Public Health. 2025;13:1512537. doi:10.3389/fpubh.2025.1512537.
11. Lockwood A, Newman DS, Mossing KW, Glubzinski A, Cohen E. Human versus machine: A comparative analysis of qualitative coding by humans and ChatGPT‑4. Sch Psychol. Published online October 20, 2025. doi:10.1037/spq0000715.
12. Castellanos A, Jiang H, Gomes P, Vander Meer D, Castillo A. Large language models for thematic summarization in qualitative healthcare research: Comparative analysis of model and human performance. JMIR AI. 2025;4:e64447. doi:10.2196/64447.
13. Kon MHA, Pereira MJ, De Castro Molina JAD, Yip VCH, Abisheganaden J, Yip WF. Unravelling ChatGPT’s potential in summarising qualitative in‑depth interviews. Eye. 2025;39:354–358. doi:10.1038/s41433-024-03419-0.
14. Lund‑Tonnesen M, Vahr Lauridsen S, Rosenberg J. Evaluating Microsoft Copilot in qualitative health research: Accurate for manifest content coding but limited in latent interpretation. Cureus. 2025;17(10):e95719. doi:10.7759/cureus.95719.
15. Goldberg EM, Macis M, Bounds M, Picazo JG, Nicholas LH. Free‑text responses in a nationally representative experimental survey about end‑of‑life care choices: ChatGPT‑4o‑assisted qualitative analytical study. JMIR Aging. 2025;8:e76335. doi:10.2196/76335.
16. Kermansaravi M, Cohen RV. Clinical obesity through the lens of context‑aware large language models. Obes Surg. 2025;35:5247‑5255. doi:10.1007/s11695-025-08341-2.
17. Chubb LA, Jackson S, Naseer B, Matthews M. To leave or stay? Influences on early exit and completion in a New Zealand residential drug rehabilitation service. Qual Health Res. 2025;0(0):1‑16. doi:10.1177/10497323251367177.
18. Parameswaran V, Bernard J, Bernard A, Deo N, Tsung S, Lyytinen K, et al. Evaluating large language models and retrieval‑augmented generation enhancement for delivering guideline‑adherent nutrition information for cardiovascular disease prevention: Cross‑sectional study. J Med Internet Res. 2025;27:e78625. doi:10.2196/78625.
19. Hairston JM, Ranjan R, Lakamana S, Spadaro A, Bozkurt S, Perrone J, Sarker A. Automating inductive thematic analyses of health content using large language models: A proof‑of‑concept study using social media data. JAMIA Open. 2025;8(5):ooaf102. doi:10.1093/jamiaopen/ooaf102.
20. Vikan M, Aryan R, Kannelønning MS, Riegler D, Danielsen S, Alexander SO. Reflecting on LLM support in reflexive thematic analysis: An exploratory study. Qual Health Res. 2025;0(0):1‑15. doi:10.1177/10497323251365211.
21. Jeminiwa RN, Popielaski C, King A. Exploring young adults' experiences and beliefs in asthma medication management: Pilot qualitative study comparing human and multiple AI thematic analyses. JMIR Form Res. 2025;9:e69892. doi:10.2196/69892.
22. Misgav K, Neufeld‑Kroszynski G, Palombo M, Karnieli‑Miller O. Human analysis vs. artificial intelligence: Analyzing qualitative medical students’ narratives. Qual Health Res. 2025;0(0):1‑13. doi:10.1177/10497323251359445.
23. Morgan DL. Query‑based analysis: A strategy for analyzing qualitative data using ChatGPT. Qual Health Res. 2025. doi:10.1177/10497323251321712.
24. Beegle S, Gomez LA, Blackard JT, et al. HIV prevention and treatment information from four artificial intelligence platforms: A thematic analysis. AIDS Behav. 2025;29(11):3394‑3403. doi:10.1007/s10461-025-04786-9.
25. Shanwetter Levit N, Saban M. When investigator meets large language models: A qualitative analysis of cancer patient decision‑making journeys. npj Digit Med. 2025;8:336. doi:10.1038/s41746-025-01747-3.
26. Liu X, He L, Alanazi E, et al. Assessing the accuracy and explainability of using ChatGPT to evaluate the quality of health news. BMC Public Health. 2025;25:2038. doi:10.1186/s12889-025-23206-0.
27. Yue Y, Liu D, Lv Y, Hao J, Cui P. A practical guide and assessment on using ChatGPT to conduct grounded theory: Tutorial. J Med Internet Res. 2025;27:e70122. doi:10.2196/70122.
28. Muasher‑Kerwin C, Hughes MC, Foster ML, Al Azher I, Alhoori H. Exploring large language models for summarizing and interpreting an online brain tumor support forum. Digit Health. 2025;11:20552076251337345. doi:10.1177/20552076251337345.
29. Young A, Omosun F. A comparative analysis of CDC and AI‑generated health information using computer‑aided text analysis. J Commun Healthc. 2025;3:205‑216. doi:10.1080/17538068.2025.2487378.
30. Nair RAS, Hartung M, Heinisch P, et al. Summarizing online patient conversations using generative language models: Experimental and comparative study. JMIR Med Inform. 2025;13:e62909. doi:10.2196/62909.
31. Keating C, Marcus SC, Bowden CF, Worsley D, Doupnik SK. Artificial intelligence and qualitative analysis of emergency department telemental health care implementation survey. Telemed J E Health. 2025;31(7):821‑828. doi:10.1089/tmj.2024.0555.
32. Abroms LC, Yousefi A, Wysota CN, Wu T‑C, Broniatowski DA. Assessing the adherence of ChatGPT chatbots to public health guidelines for smoking cessation: Content analysis. J Med Internet Res. 2025;27:e66896. doi:10.2196/66896.
33. Stage MA, Creamer MM, Ruben MA. Having providers who are trained and have empathy is life‑saving: Improving primary‑care communication through thematic analysis with ChatGPT and human expertise. PEC Innov. 2024;6:100371. doi:10.1016/j.pecinn.2024.100371.
34. Kondo T, Miyachi J, Jönsson A, Nishigori H. A mixed‑methods study comparing human‑led and ChatGPT‑driven qualitative analysis in medical education research. Nagoya J Med Sci. 2024;86(4):620‑644. doi:10.18999/nagjms.86.4.620.
35. Beck S, Kuhner M, Haar M, et al. Evaluating the accuracy and reliability of AI chatbots in disseminating the content of current resuscitation guidelines: A comparative analysis. Scand J Trauma Resusc Emerg Med. 2024;32:95. doi:10.1186/s13049‑024‑01266‑2.
36. Deiner MS, Honcharov V, Li J, Mackey TK, Porco TC, Sarkar U. Large language models can enable inductive thematic analysis of a social media corpus in a single prompt: Human validation study. JMIR Infodemiology. 2024;4:e59641. doi:10.2196/59641.
37. Wosny M, Hastings J. Applying large language models to interpret qualitative interviews in healthcare. Stud Health Technol Inform. 2024;316:791‑795. doi:10.3233/SHTI240530.
38. Li KD, Fernandez AM, Schwartz R, Rios N, Carlisle MN, Amend GM, et al. Comparing GPT‑4 and human researchers in health care data analysis: Qualitative description study. J Med Internet Res. 2024;26:e56500. doi:10.2196/56500.
39. Mathis WS, Zhao S, Pratt N, Weleff J, De Paoli S. Inductive thematic analysis of healthcare qualitative interviews using open‑source LLMs: How does it compare to traditional methods? Comput Methods Programs Biomed. 2024;255:108356. doi:10.1016/j.cmpb.2024.108356.
40. Bijker R, Merkouris SS, Dowling NA, Rodda SN. ChatGPT for automated qualitative research: Content analysis. J Med Internet Res. 2024;26:e59050. doi:10.2196/59050.
41. Leas EC, Ayers JW, Desai N, Dredze M, Hogarth M, Smith DM. Using large language models to support content analysis: A case study of ChatGPT for adverse event detection. J Med Internet Res. 2024;26:e52499. doi:10.2196/52499.
42. Bragazzi NL, Garbarino S. Assessing the accuracy of generative conversational artificial intelligence in debunking sleep health myths: Mixed‑methods comparative study with expert analysis. JMIR Form Res. 2024;8:e55762.
